# The NKL-code for innate lymphoid cells reveals deregulated expression of NKL homeobox genes HHEX and HLX in anaplastic large cell lymphoma (ALCL)

**DOI:** 10.18632/oncotarget.27683

**Published:** 2020-08-25

**Authors:** Stefan Nagel, Claudia Pommerenke, Roderick A.F. MacLeod, Corinna Meyer, Maren Kaufmann, Hans G. Drexler

**Affiliations:** ^1^Department of Human and Animal Cell Lines, Leibniz Institute, DSMZ – German Collection of Microorganisms and Cell Cultures, Braunschweig, Germany

**Keywords:** homeobox, NKL-code, T-ALL, LL-100, Th17

## Abstract

NKL homeobox genes encode developmental transcription factors and display an NKL-code according to their physiological expression pattern in hematopoiesis. Here, we analyzed public transcriptome data from primary innate lymphoid cells (ILCs) for NKL homeobox gene activities and found that ILC3 expressed exclusively HHEX while in ILC1 and ILC2 these genes were silenced. Deregulation of the NKL-code promotes hematopoietic malignancies, including anaplastic large cell lymphoma (ALCL) which reportedly may derive from ILC3. Accordingly, we analyzed NKL homeobox gene activities in ALCL cell lines and investigated their role in this malignancy. Transcriptome analyses demonstrated low expression levels of HHEX but powerfully activated HLX. Forced expression of HHEX in ALCL cell lines induced genes involved in apoptosis and ILC3 differentiation, indicating tumor suppressor activity. ALCL associated NPM1-ALK and JAK-STAT3-signalling drove enhanced expression of HLX while discounting HHEX. Genomic profiling revealed copy number gains at the loci of HLX and STAT3 in addition to genes encoding both STAT3 regulators (AURKA, BCL3, JAK3, KPNB1, NAMPT, NFAT5, PIM3, ROCK1, SIX1, TPX2, WWOX) and targets (BATF3, IRF4, miR135b, miR21, RORC). Transcriptome data of ALCL cell lines showed absence of STAT3 mutations while MGA was mutated and downregulated, encoding a novel potential STAT3 repressor. Furthermore, enhanced IL17F-signalling activated HLX while TGFbeta-signalling inhibited HHEX expression. Taken together, our data extend the scope of the NKL-code for ILCs and spotlight aberrant expression of NKL homeobox gene HLX in ALCL. HLX represents a direct target of ALCL hallmark factor STAT3 and deregulates cell survival and differentiation in this malignancy.

## INTRODUCTION

Lymphocytes are white blood cells and include those possessing adaptive immunity (B-cells and T-cells) and cells which lack this specificity and belong to the innate immune system (NK-cells and innate lymphoid cells: ILCs). Their development begins in the bone marrow with the hematopoietic stem cell (HSC)-derived common lymphoid progenitor (CLP). Downstream of CLPs are B-cell progenitors (BCP), early T-cell progenitors (ETP) and common innate lymphoid progenitors (CILP) which generate NK-cells and ILCs. These differentiation processes are mainly regulated at the transcriptional level [1–3]. Thus, master genes encoding transcription factors (TF) are reported for B-cells (PAX5, TCF3), T-cells (BCL11B, GATA3) and NK-cells (NFIL3, EOMES, ID2) [4–6]. T-helper (TH) cells and ILCs are each classified into subgroups reflecting their diverse cytokine profiles and which master genes regulate their differentiation: TH1 (IFNG and TBX21), TH2 (IL4 and GATA3), TH17 (IL17A/F and STAT3), Treg (TGFb and FOXP3), ILC1 (GZMB and TBX21), ILC2 (IL13 and GATA3, GFI1), and ILC3 (CSF2 and AHR, TCF7, RORC) [7]. Interestingly, extended gene signatures of the ILCs resemble that of the TH cells: ILC1 expression patterns resemble TH1, ILC2 TH2, and ILC3 TH17 cells [8].

NKL homeobox genes represent a 48 gene-strong subclass of the ANTP homeobox gene class and encode TFs which regulate fundamental processes in development [9, 10]. Accordingly, particular NKL homeobox genes including HHEX and HLX are expressed in the course of hematopoiesis, exerting major impacts on cell lineage decisions [11, 12]. They are expressed in HSCs and progenitors of myeloid and lymphoid lineages and remain active in most mature myeloid cells. In lymphoid cells, HHEX expression persists only in memory B-cells while HLX is silent in all types of lymphocytes [13–15]. Accordingly, knockout studies in mice demonstrated a prominent role of HHEX in lymphoid development including B-cells, T-cells, and NK-cells [12]. In contrast, continous expression of HLX in murine lymphopoiesis disturbed the development of B-cells and T-cells [11]. Thus, HHEX and HLX are fundamentally involved in myeloid and lymphoid differentiation but show significant differences in their expression and function.

We have identified eleven members of the NKL homeobox gene subclass specifically expressed in early hematopoiesis, lymphopoiesis and myelopoiesis and termed this signature NKL-code [13–15]. Alterations of this code via ectopic activation of non-code members or deregulation of code-members have been described in both lymphoid and myeloid malignancies [13–15]. Their oncogenic impact in hematopoietic tumors has been reported for the first time in T-cell acute lymphoid leukemia (T-ALL) in which NKL non-code member TLX1 is activated by chromosomal translocation juxtaposed to a T-cell receptor gene [16]. MSX1 represents a code-member and is normally expressed in CLPs, BCPs and NK-cells [13, 14, 17]. Aberrantly enhanced activity of MSX1 has been described in T-ALL and its aberrant suppression in NK-cell malignancies, thus performing as an oncogene and tumor suppressor gene, respectively [17, 18].

Anaplastic large cell lymphoma (ALCL) belongs to the group of CD30-positive cytotoxic T-cell and NK-cell lymphomas [19, 20]. This malignancy is characterized by the presence of chromosomal rearrangement t(2;5)(p23;q35) which generates a fusion gene consisting of NPM1 and ALK [21]. The subsequently enhanced activity of the ALK encoded tyrosine kinase drives the JAK-STAT3-pathway which in turn deregulates several oncogenic downstream targets [22]. ALCLs lacking the fusion gene NPM1-ALK possess activating mutations of STAT3 instead, highlighting the role of aberrant STAT3 activity in this malignancy [23]. However, deregulated and mutated STAT3 is not restricted to ALCL and plays an oncogenic role in other types of T- and NK-cell lymphomas as well [24, 25]. Aberrant activities of AP1 TFs including JUN, JUNB and BATF3 are an additional ALCL hallmark [26]. Moreover, BATF3 is a target gene of STAT3 [27], demonstrating close regulatory relationships between these oncogenes in ALCL. Expression profiling analysis indicated that primary ALCL cells express several TH17 signature genes [28]. According to the molecular similarity of TH17 cells and ILC3, Schleussner and coworkers postulated that ALCL subtypes may derive from ILC3, possibly representing their cell of origin [29].

Here, we screened NKL homeobox gene activities in primary ILCs, thereby extending the NKL-code to this type of lymphocyte. In addition we analyzed deregulated NKL homeobox genes HHEX and HLX in ALCL cell lines and their relation to ILC3/TH17 cell differentiation.

## RESULTS

### ILC3 express NKL homeobox gene HHEX

Analysis of gene activities in primary ILCs was performed using public transcriptome dataset GSE112591 generated from purified ILC subsets which were isolated from the peripheral blood of healthy donors [30]. We found differential expression of several known ILC markers including cytokine and master factor encoding genes in addition to novel candidates in ILC1 (GZMB and TBX21, PRDM1), ILC2 (IL13 and GATA3, GFI1, LMO4), and ILC3 (CSF2 and TCF7, AHR, NFIL3, IL1R1, IL2RA, TIMP1, ID2, CARD19, BHLHE40), supporting the validity of these data (Supplementary Table 1, Supplementary Figure 1A–1C) [7].

**Figure 1:**
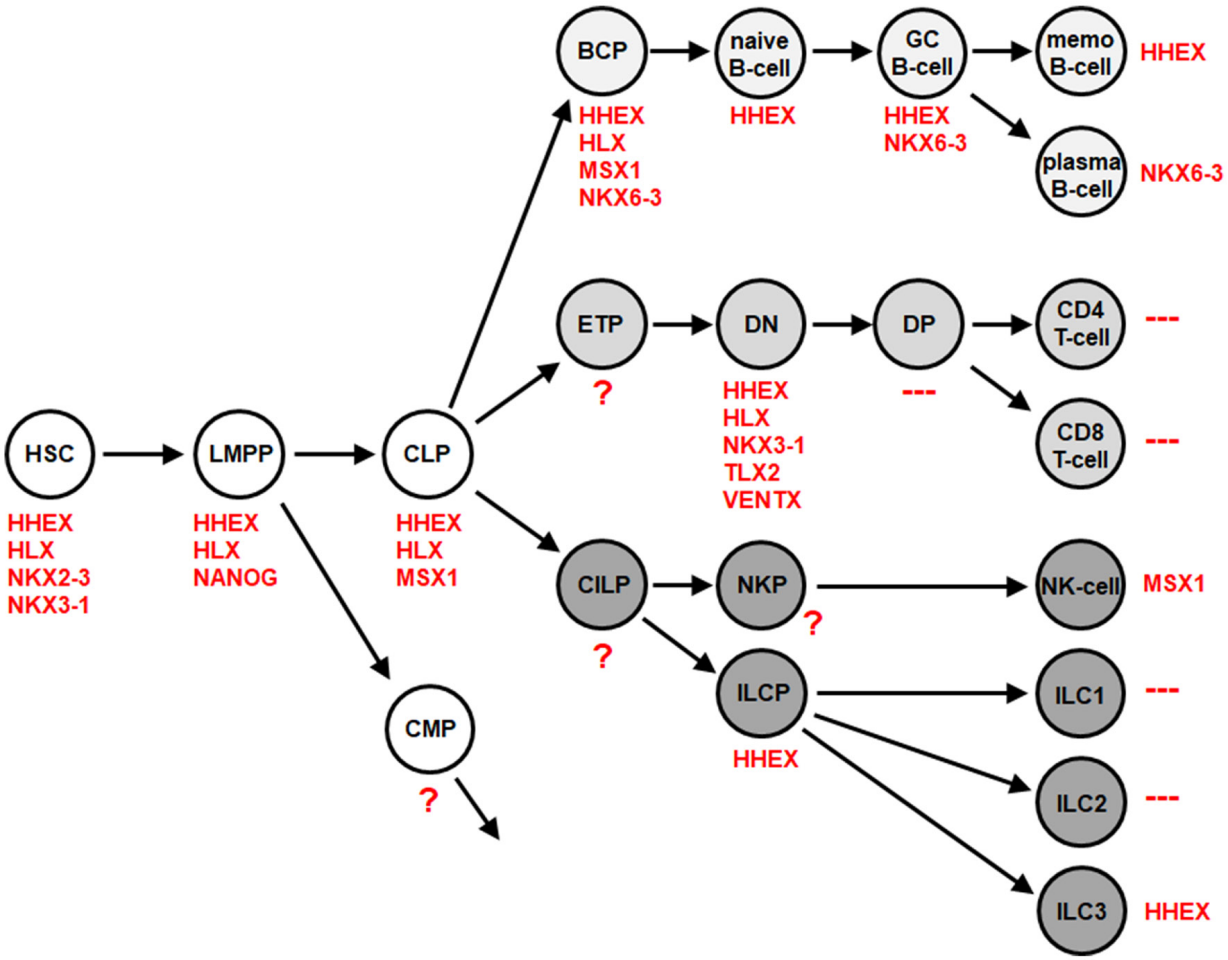
NKL-code for the lymphoid system. This diagram depicts developing and mature hematopoietic cells and indicates the NKL homeobox genes activated therein comprising the NKL-code. Abbreviations are indicated in the text. According to transcriptome dataset GSE112591, ILC3 express HHEX while ILC1 and ILC2 are negative for all NKL homeobox genes.

Subsequent expression analysis of the complete group of 48 NKL homeobox genes in this dataset identified HHEX exclusively in ILC3 while ILC1 and ILC2 were negative for this homeobox gene subclass (Supplementary Table 1, Supplementary Figure 1D). Exclusively high expression levels of HHEX were also detected in ILC3 using dataset GSE124474 generated from cells isolated from both peripheral blood and tonsils (Supplementary Table 2, Supplementary Figure 1D) [31]. ILC3 isolated from peripheral blood may represent ILC precursors (ILCP) which are able to generate all ILC subtypes including ILC3 [32, 33]. However, expression data of ILCP also showed high HHEX expression (Supplementary Table 3, Supplementary Table 4, Supplementary Figure 1D). Thus, both ILCP and derived ILC3 expressed exclusively elevated NKL homeobox gene HHEX independent of their source. Of note, analysis of NKL homeobox genes in primary immune cells isolated from the peripheral blood using transcriptome dataset GSE107011 [34], indicated absence of HHEX expression in ILC3-related TH17 cells (Supplementary Figure 1E). Thus, the related ILC3 and TH17 cells differ in HHEX activity. Then, we integrated these NKL homeobox gene expression results into the NKL-code to extend this signature for the lymphoid system (Figure 1). Accordingly, mature B-cells express HHEX or NKX6-3 while T-cells are negative for NKL homeobox genes [13, 14]. Among innate lymphocytes NK-cells express MSX1 and ILC3 HHEX while ILC1 and ILC2 lack any activity of NKL homeobox genes [17].

### HHEX regulates apoptosis and ILC3 differentiation in ALCL cell lines

Primary ALCL cells show a TH17-like gene signature and may originate from ILC3 cells [28, 29]. Selected transcriptome data of 100 leukemia/lymphoma cell lines (dataset LL-100) demonstrated clustering of ALCL cell lines in comparison to congeners derived from NK-cell malignancies, T-ALL and Hodgkin lymphoma (HL) (Supplementary Figure 2A). Furthermore, these data showed significant expression levels of ILC3-specific marker genes in ALCL cell lines including BHLHE40, CARD19, ID2, IL17F, IL1R, IL2RA, IL23R, NFIL3, RORC and TIMP1 (Supplementary Figure 2B). Thus, these ALCL cell lines represent a distinct group of tumor cell lines which express multiple ILC3-markers.

**Figure 2:**
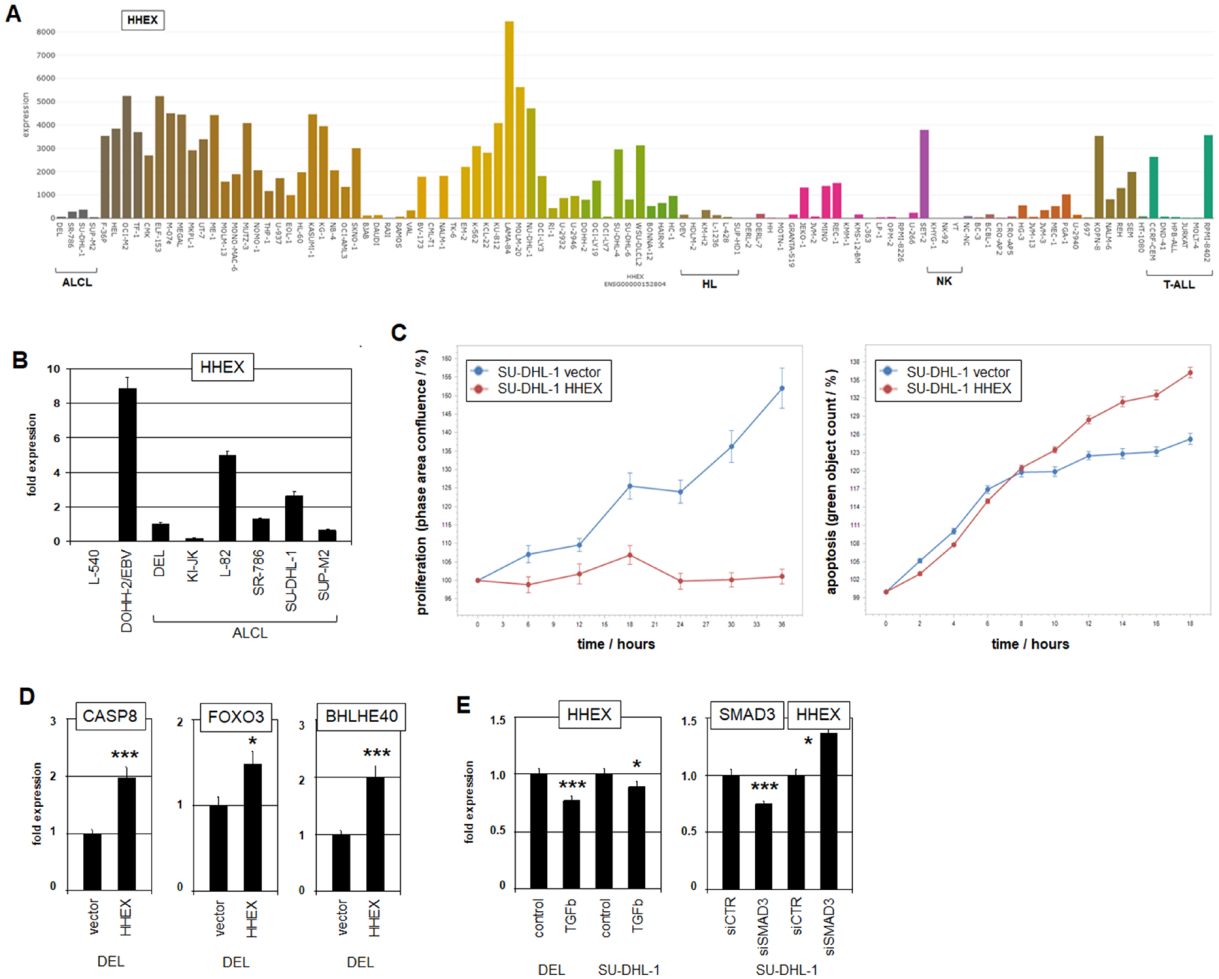
Expression, function and regulation of HHEX. (**A**) HHEX expression in 100 hematopoietic tumor cell lines according to the LL-100 transcriptome dataset (PRJEB30312). Please note the low expression levels in ALCL cell lines. Cell lines derived from HL, NK-cell malignancies and T-ALL are indicated. (**B**) RQ-PCR analysis of HHEX in ALCL and control cell lines L-540 and EBV-positive DOHH-2. (**C**) Live-cell-imaging analyses of SU-DHL-1 cells after forced expression of HHEX. The data show that HHEX inhibits proliferation (left) and activates apoptosis (right). (**D**) RQ-PCR analysis of potential HHEX target genes CASP8 (left), FOXO3 (middle) and BHLHE40 (right) in ALCL cell line DEL. Fold expression levels are indicated in relation to the vector control. (**E**) RQ-PCR analysis of ALCL cell lines DEL and SU-DHL-1 for HHEX after treatment with TGFbeta (left). RQ-PCR analysis of ALCL cell line SU-DHL-1 after siRNA-mediated knockdown of SMAD3 for SMAD3 and HHEX (right).

ALCL patient samples have been shown to overexpress several NKL homeobox genes, indicating an oncogenic role for these genes in this malignancy [35]. However, ALCL cell lines DEL, SR-786, SU-DHL-1 and SUP-M2 expressed only low transcript levels of HHEX in comparison to controls (Figure 2A, 2B), discounting oncogenic activity for this ILC3-specific gene in these cells. Consistent with this view, forced expression of HHEX in ALCL cell line SU-DHL-1 inhibited proliferation and induced apoptosis as analyzed by live-cell-imaging (Figure 2C, Supplementary Figure 3A).

Expression profiling data for SU-DHL-1 cells driven to overexpress HHEX revealed activation of the p53-pathway and inhibition of the NFkB-pathway (Supplementary Table 5, Supplementary Figure 3B), yielding an explanation for HHEX-mediated apoptosis. RQ-PCR analysis of selected candidates demonstrated activation of genes involved in apoptosis (CASP8 and FOXO3) and differentiation towards ILC3 (BHLHE40) (Figure 2D). Accordingly, in a genomic screening we identified potential HHEX binding sites in the regulatory upstream regions of CASP8 (CAATTAAG at -3129 bp), FOXO3 (CAATTAAC at -4699 bp) and BHLHE40 (CAATTAAA at -4724 bp), suggesting direct regulation [36]. Moreover, analysis of expression profiling data from selected ALCL (GSE19069) and peripheral T-cell lymphoma patients (GSE6338) showed correlated activities of HHEX, CASP8, FOXO3 and BHLHE40 (Supplementary Figure 4), supporting this regulatory connection. Thus, HHEX activity is associated with apoptosis and differentiation, indicating tumor suppressor activity in ALCL.

HHEX has been described as downregulated target of the TGFbeta-SMAD-pathway in both prostate cells and regulatory T-cells [37, 38]. Accordingly, treatment of ALCL cell lines DEL and SU-DHL-1 with TGFbeta reduced HHEX expression and siRNA-mediated knockdown of TGFbeta-effector SMAD3 resulted in increased expression levels in DEL (Figure 2E). Thus, this pathway plays an inhibitory role for HHEX regulation in ALCL as well. However, the TGFbeta-signalling pathway has not been implicated in the pathogenesis of ALCL so far, although ALCL cell lines showed prominent expression levels of HHEX inhibitor SMAD3 (Supplementary Figure 2B).

### Aberrant expression of HLX in ALCL

**Figure 3:**
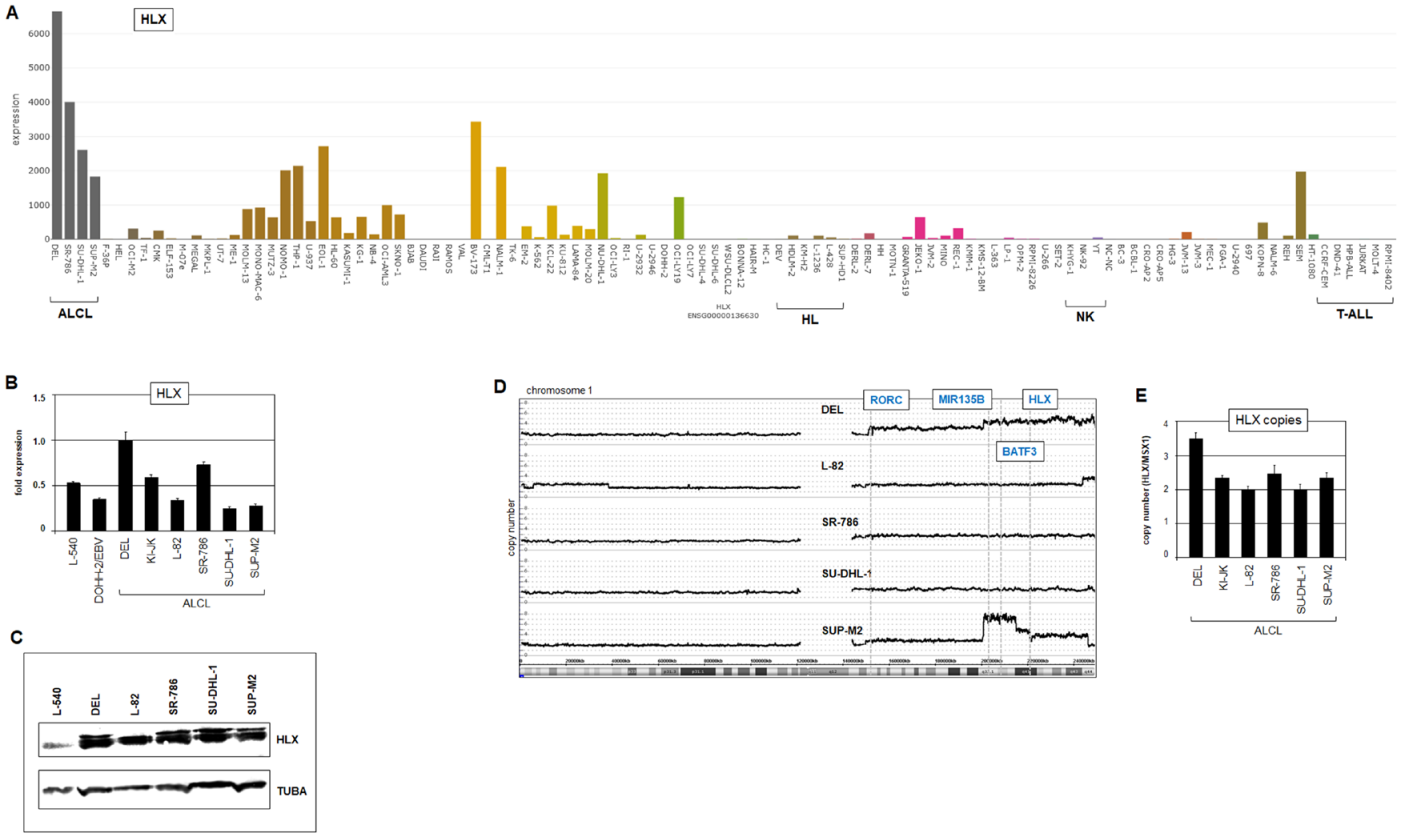
Expression and genomic aberrations of HLX. (**A**) HLX expression in 100 hematopoietic tumor cell lines according to the LL-100 transcriptome dataset (PRJEB30312). (**B**) RQ-PCR analysis of HLX in ALCL cell lines in addition to control cell lines L-540 and EBV-positive DOHH-2. (**C**) Western blot analysis of HLX in ALCL cell lines. TUBA served as control. (**D**) Genomic profiling analysis of ALCL cell lines revealed copy number alterations in chromosome 1 including gains at 1q41 containing HLX. (**E**) Genomic RQ-PCR analysis confirmed copy number gain of HLX in DEL.

Comprehensive analysis of 48 NKL homeobox gene activities in ALCL cell lines DEL, SR-786, SU-DHL-1 and SUP-M2 using transcriptome dataset LL-100 identified strong expression of HLX in all four cell lines (Figure 3A). In addition, elevated expression levels were detected of MSX1 in DEL and SU-DHL-1, and of NKX2-2 in SU-DHL-1 (Supplementary Figure 5). RQ-PCR and Western blot analyses confirmed prominent HLX activity in ALCL cell lines (Figure 3B, 3C), with DEL showing the highest level. Overexpression of HLX has been detected in subsets of ALCL patients [35], supporting the pathological relevance of its activation. Furthermore, as indicated by transcriptome data of normal immune cells, TH17 cells did not express HLX (Supplementary Figure 1F), underlining its aberrant activity in ALCL. In the following, ALCL cell lines served as models to study mechanisms of deregulation and the functional impacts of NKL homeobox gene HLX in this malignancy.

### Chromosomal and genomic analyses of ALCL cell lines

To look for potential chromosomal alterations responsible for deregulation of HLX (and HHEX) in ALCL cell lines we performed karyotyping (Supplementary Figure 6A) and genomic profiling (Supplementary Figure 7) of DEL, L-82, SR-786, SU-DHL-1 and SUP-M2. The karyotypes indicated that HLX (located at chromosomal position 1q41) was not targeted by chromosomal rearrangements directly. Of note, karyotyping results also excluded chromosomal rearrangements at the loci of HHEX (10q23), MSX1 (4p16) or NKX2-2 (20p11). Furthermore, t(2;5)(p23;q35) was detected in L-82, SR-786, SU-DHL-1 and SUP-M2 but not in DEL. Nevertheless, all used ALCL cell lines expressed the fusion gene NPM1-ALK as analyzed by RT-PCR (Supplementary Figure 6B), indicating the presence of a cryptic fusion in DEL masked by t(5;6)(q35;p21) in this cell line.

Generated genomic profiling data were used to identify genomic regions with altered copy numbers together with the corresponding gene loci. Detected copy number gains contained HLX in DEL (Figure 3D), and NKX2-2 in SR-786 and SU-DHL-1 while the loci of HHEX and MSX1 showed wild type configurations in all analyzed cell lines (Supplementary Figure 7). The copy number gain of the HLX locus was additionally confirmed by RQ-PCR (Figure 3E). Thus, these genomic alterations may underlie increased expression of HLX and NKX2-2 in ALCL cells.

Then, we extended this analysis and examined the activities of all genes located in genomic regions targeted by copy number alterations. Table 1 lists selected genes showing their genomic localization and reported functions. Interestingly, in addition to STAT3 itself (located at 17q21), several genes are regulatorily connected with STAT3, variously reported as activators (AURKA, BCL3, BRD2, JAK3, KPNB1, NFAT5, PIM3, ROCK1, SIX1, TPX2), inhibitors (WWOX), and targets (BATF3, HLX, IRF4, miR135b, NFATC1, RORC, VMP1/miR21). Accordingly, siRNA-mediated knockdown of STAT3 in ALCL cell line SU-DHL-1 confirmed that IRF4 and miR135b represent STAT3 target genes [40, 43] while HHEX escaped regulation by STAT3 (Supplementary Figure 8). Together, these data highlight the oncogenic impact of both copy number alterations and STAT3 activity for the pathogenesis of ALCL.

**Table 1:** Copy number alterations in ALCL cell lines

Aberration	Locus	Cell Line	Gene	Function	Ref.
gain	1q21	DEL, SUP-M2	RORC	target of **STAT3**	[39]
gain	1q32	DEL, SUP-M2	miR135b	target of **STAT3**, inhibits GATA3	[40]
gain	1q32	DEL, SUP-M2	BATF3	target of **STAT3**, activates MYC	[27]
gain	1q41	DEL, SUP-M2	HLX	target of **STAT3**, de-differentiation	[41]
loss	2q32	L-82, SU-DHL-1, SUP-M2	ITGAV	target of JUN	[42]
gain	6p25	DEL, L-82, SUP-M2	IRF4	target of **STAT3**	[43]
gain	6p21	L-82, SU-DHL-1, SUP-M2	TCF19	proliferation	[44]
gain	6p21	L-82, SUP-M2	BRD2	activates **STAT3**	[45]
gain	7q21	SU-DHL-1	CDK6	proliferation	
gain	7q22	L-82	NAMPT	activates SIRT1	[46]
gain	8q11	SU-DHL-1	ST18	?	
gain	8q24	L-82, SU-DHL-1	MYC	proliferation	
gain	9p24	L-82	DMRT1	inhibits RA-signalling	[47]
gain	9p21	SR-786, SUP-M2	LOC401497	non-coding RNA	—
gain	9p21	SR-786	ACO1	metabolic signature	[48]
loss	10p14	L-82	GATA3	target of miR135b	[40]
loss	10q25	DEL, SU-DHL-1	CASP7	apoptosis	[49]
loss	12q24	DEL	SUDS3	differentiation	[50]
gain	13q12	SR-786	MTMR6	survival	[51]
loss	14q11	L-82, SR-786, SU-DHL-1, SUP-M2	DAD1	survival	[52]
gain	14q23	SU-DHL-1	SIX1	activates **STAT3**	[53]
loss	15q26	DEL, SR-786	CHD2	chromatin modulator	
gain	16q22	SU-DHL-1	NFAT5	activates **STAT3**	[54]
loss	16q23	L-82, SU-DHL-1, SUP-M2	WWOX	inhibits JAK/**STAT3**	[55]
gain	17q21	SU-DHL-1	**STAT3**	hallmark for ALCL	[23]
gain	17q21	SU-DHL-1	KPNB1	nuclear import of **STAT3**	[56]
break, gain	17q23	DEL, SU-DHL-1, SUP-M2	VMP1/miR21	target of **STAT3**	[57]
gain	18q11	SR-786	ROCK1	activates JAK/**STAT3**	[58]
gain	18q21	SU-DHL-1	SMAD4	inhibits HHEX	[38]
loss	18q23	SU-DHL-1	NFATC1	target of **STAT3**, differentiation	[59]
gain	19p13	L-82, SUP-M2	JAK3	acitvates **STAT3** in ALCL	[60]
gain	19q13	SU-DHL-1	BCL3	activates **STAT3**	[61]
gain	20p11	SR-786, SU-DHL-1	NKX2-2	de-differentiation	[35]
gain	20q11	L-82, SR-786, SU-DHL-1	BCL2L1	survival	
gain	20q11	L-82, SR-786, SU-DHL-1	TPX2	activates AURKA	[62]
gain	20q13	SR-786, SU-DHL-1	AURKA	activates JAK2 and **STAT3**	[63]
gain	22q13	DEL, SR-786, SU-DHL-1	PIM3	activates **STAT3**	[64]

### STAT3 activates HLX in ALCL directly

**Figure 4:**
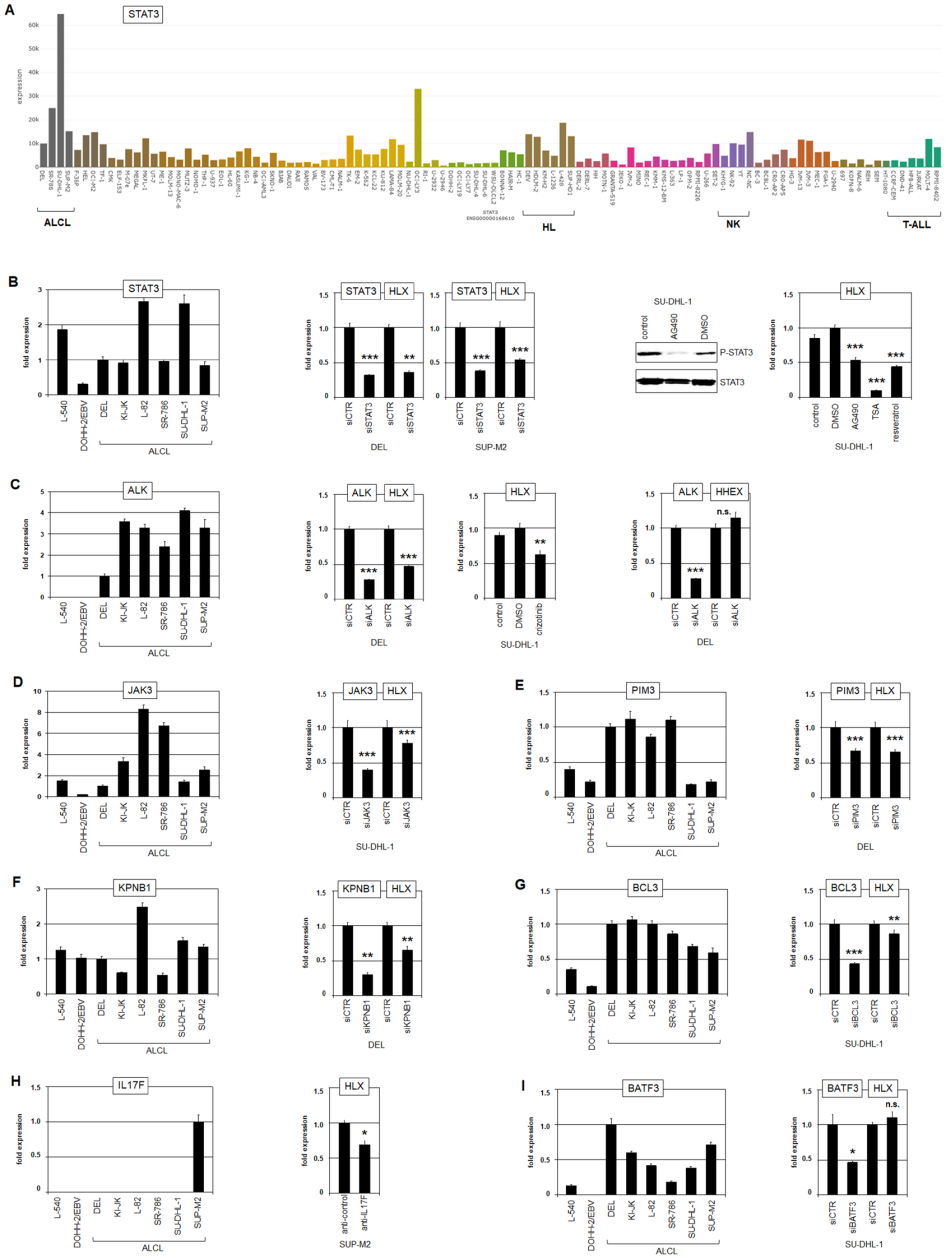
STAT3 activates HLX. (**A**) STAT3 expression in 100 hematopoietic tumor cell lines according to the LL-100 transcriptome dataset (PRJEB30312). (**B**) RQ-PCR analysis of STAT3 in ALCL cell lines in addition to control cell lines L-540 and EBV-positive DOHH-2 (left). SiRNA-mediated knockdown of STAT3 in DEL and SUP-M2 resulted in reduced HLX expression (middle). Pharmacological inhibition of STAT3-phosphorylation by AG490 was confirmed by Western blot and resulted in reduced HLX expression as analyzed by RQ-PCR (right). Inhibition of deacetylation by TSA and resveratrol resulted in reduced HLX expression as well (right). (**C**) RQ-PCR analysis of ALK in ALCL cell lines in addition to control cell lines L-540 and EBV-positive DOHH-2 (left). SiRNA-mediated knockdown of ALK in DEL resulted in reduced HLX expression (middle) but not of HHEX (right). Pharmacological inhibition of ALK by crizotinib resulted in reduced HLX expression (middle). (**D**–**G**) Analysis of the impacts of additional STAT3 activators on HLX expression in ALCL cell lines by RQ-PCR and siRNA-mediated knockdown: JAK3 (D), PIM3 (E), KPNB1 (F), and BCL3 (G). (**H**) RQ-PCR analysis of IL17F indicated enhanced expression in ALCL cell line SUP-M2 (left). Treatment of SUP-M2 cells with inhibitory antibody directed against IL17F resulted in reduced HLX expression (right). (**I**) RQ-PCR analysis of BATF3 in ALCL cell lines (left). SiRNA-mediated knockdown of BATF3 showed no significant effect on HLX expression (right).

Due to copy number gains, the expression level of STAT3 was prominently elevated in ALCL cell line SU-DHL-1 as shown by LL-100 transcriptome data and RQ-PCR analysis (Figure 4A, 4B). L-82 cells expressed high STAT3 levels as well, indicating that additional oncogenic factors enhance STAT3 transcription in this malignancy. STAT3 has been shown to activate HLX expression in cell lines derived from HL (L-540) and EBV-positive diffuse large B-cell lymphoma (DLBCL, DOHH-2/EBV) [41, 65]. To analyze the impact of STAT3 on HLX in ALCL we performed siRNA-mediated knockdown and pharmacological inhibition by AG490. These results clearly showed that HLX is activated by STAT3 in ALCL cell lines as well (Figure 4B). Furthermore, deacetylase-inhibitors TSA and resveratrol also inhibited HLX expression (Figure 4B), indicating that reported acetylation-mediated nuclear export of STAT3 was also responsible for HLX suppression in treated ALCL cell lines [65]. ChIP-seq data for STAT3 have been generated for ALCL cell line SU-DHL-1 [66], demonstrating binding of STAT3 in the regulatory upstream and downstream regions of HLX (Supplementary Figure 9A). The upstream position has been functionally confirmed in a HL cell line [65], and the downstream position contains a consensus STAT3 binding site (Supplementary Figure 9B), supporting a direct regulatory impact. Furthermore, these ChIP-seq data confirmed STAT3 regulation of IRF4 and miR135b (see above) in addition to BATF3, VMP1, RORC and IL17F and show lack of STAT3 binding to sequences at HHEX, MSX1 and NKX2-2 (Supplementary Figure 9A).

### Various factors activate HLX via STAT3 in ALCL

ALK is activated in ALCL cell lines by recurrent chromosomal aberrations, notably t(2;5)(p23;q35) which results in a fusion with NPM1 and high ectopic ALK expression levels (Supplementary Figures 5 and 6, Figure 4C). SiRNA-mediated knockdown of ALK and its pharmacological inhibition by crizotinib demonstrated that HLX was activated by NPM1-ALK which has been shown to drive the JAK-STAT3-pathway (Figure 4C) [22]. Expression profiling analysis of ALCL patient samples revealed significant correlations between HLX, ALK and STAT3 (Supplementary Figure 10), underlining the clinical relevance of this regulatory connection. Consistent with our previous results HHEX was not regulated by ALK (Figure 4C). Furthermore, siRNA-mediated knockdown of genes targeted by copy number gains including JAK3, PIM3, KPNB1 and BCL3 demonstrated that these reported STAT3-activators support HLX expression in ALCL as well (Figure 4D–4G).

IL17F represents a central marker for the gene signature of TH17-cells [67]. The IL17F-signalling pathway activates several downstream factors including STAT3 [68], drawing attention to its potential role in HLX regulation. We recognized high expression levels of IL17F RNA in HLX expressing ALCL cell line SUP-M2 (Supplementary Figure 2, Figure 4H). Accordingly, ELISA analysis detected high amounts of about 30 ng/ml IL17F protein in the supernatant of SUP-M2 while SU-DHL-1 tested negative (Supplementary Figure 11A). In cutaneous T-cell lymphoma (CTCL) cell lines mutated SOCS1 boosted IL17F expression via the IL2-JAK3-STAT5 pathway [69]. However, LL-100 transcriptome data discounted mutations in JAK3, SOCS1 and STAT5, and showed no conspicuous expression levels of these genes in SUP-M2. Furthermore, IL17F is located at 6p12 which escapes copy number alterations in this cell line (Supplementary Figure 7). In contrast, SUP-M2 contains in addition to t(2;5)(p23;q35) the chromosomal rearrangement t(6;6)(q27;p11) which might underlie IL17F activation (Supplementary Figure 6). However, FISH analysis excluded a nearby breakpoint at IL17F in SUP-M2 (Supplementary Figure 11B), leaving open the mechanism underlying its enhanced activation. Nevertheless, treatment of SUP-M2 cells with inhibitory IL17F antibody reduced transcription of HLX (Figure 4H), demonstrating that IL17F-signalling mediated activation of HLX expression in ALCL.

Activating mutations of STAT3 have been frequently detected in NK- and T-cell lymphomas including ALK-negative ALCL [24, 25]. Inactivating mutations of MGA are present in T-cell and NK-cell lymphoma patients which carry no STAT3 mutation [25], suggesting complementary effects of these genes. Fittingly, analyzing the LL-100 dataset we detected mutations in MGA but not in STAT3 in all ALCL cell lines (Supplementary Figure 12A). Furthermore, MGA expression levels were significantly reduced in ALCL and NK-cell lines as well as in ALCL patients (Supplementary Figure 12B, 12C), supporting its tumor suppressor status in these malignancies. Interestingly, analysis of public ChIP-seq data for MGA in cell line 293 showed binding of this TF at the transcriptional start site of STAT3 (Supplementary Figure 12D). MGA recruits polycomb repressor complex (PRC)6.1, resulting in target gene silencing [70]. Therefore, these findings indicate that mutation and downregulation of MGA in ALCL prevents inhibition of STAT3 transcription by PRC6.1.

Aberrant activity of AP1 factors including BATF3, JUN and JUNB represents another hallmark of ALCL [26, 29]. Accordingly, transcriptome data demonstrated elevated expression levels of these genes in ALCL cell lines (Supplementary Figure 5). Furthermore, our genomic profiling data show copy number gains of BATF3 (at 1q32) in DEL and SUP-M2 (Supplementary Figure 7, Table 1), supporting their oncogenic role in this malignancy. However, siRNA-mediated knockdown of BATF3 in SU-DHL-1 showed no altered expression levels of HHEX and HLX (Figure 4I), indicating that AP1 factors are not significantly involved in the regulation of these NKL homeobox genes. Thus, HLX is directly regulated by oncogenic STAT3 which is in turn activated by various deregulated factors in ALCL including NPM1-ALK, JAK3, PIM3, KPNB1, BCL3, IL17F and MGA.

### HLX deregulates apoptosis and differentiation in ALCL

**Figure 5:**
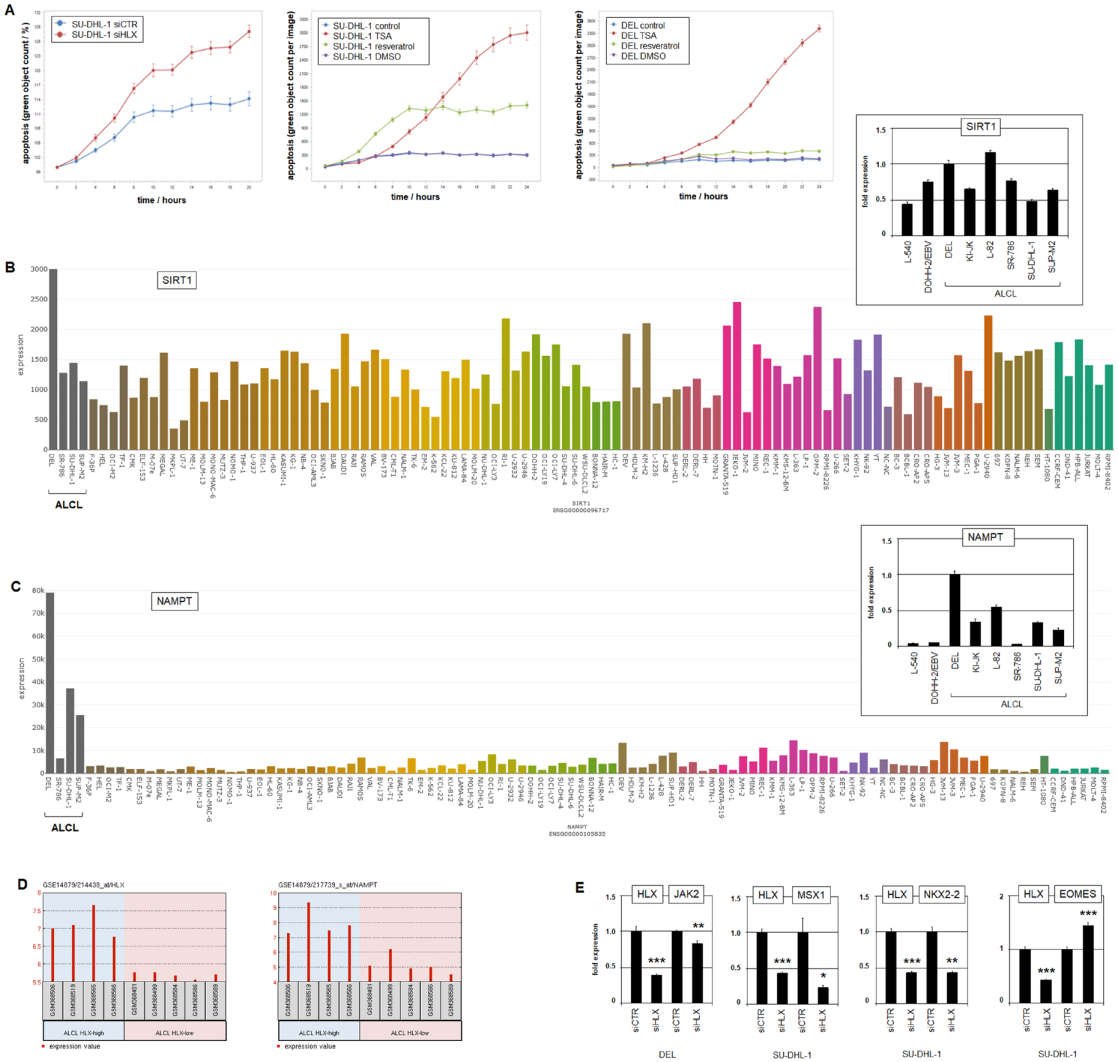
Function of HLX. (**A**) Live-cell-imaging analysis of SU-DHL-1 cells after siRNA-mediated knockdown of HLX, showing an apoptotic effect (left). Live-cell-imaging analysis of SU-DHL-1 (middle) and DEL cells (right) after treatment with deacetylase-inhibitors TSA and resveratrol, showing apoptotic effects. Of note, DEL is less sensitive to treatment with resveratrol. (**B**) SIRT1 expression in 100 hematopoietic tumor cell lines according to the LL-100 transcriptome dataset (PRJEB30312). RQ-PCR analysis of SIRT1 in ALCL cell lines and control cell lines L-540 and EBV-positive DOHH-2 (insert right). Note elevated SIRT1 levels in DEL. (**C**) NAMPT expression in 100 hematopoietic tumor cell lines according to the LL100 transcriptome dataset (PRJEB30312). RQ-PCR analysis of NAMPT in ALCL cell lines in addition to control cell lines L-540 and EBV-positive DOHH-2 (insert right). Note elevated NAMPT levels in ALCL cell lines including DEL. (**D**) Expression profiling data of ALCL patients (dataset GSE14879), showing significantly correlated gene activities of HLX (left) and NAMPT (right). (**E**) RQ-PCR analysis of HLX target genes JAK2, MSX1, NKX2-2 and EOMES after siRNA-mediated knockdown of HLX in ALCL cell lines DEL and SU-DHL-1.

To study functional consequences of HLX activity in ALCL we performed live-cell-imaging of SU-DHL-1 cells after siRNA-mediated knockdown of HLX. These experiments showed increased apoptosis, indicating that HLX effected survival in ALCL cells (Figure 5A, Supplementary Figure 13A). Treatment of SU-DHL-1 cells with deacetylase-inhibitors TSA and resveratrol induced apoptosis as well (Figure 5A, Supplementary Figure 13B), suggesting that increased levels of acetylated STAT3 mediated this effect at least partly via reduced activation of HLX [65]. Interestingly, DEL was much less sensitive to SIRT1-inhibitor resveratrol than SU-DHL-1. This observation might be related to enhanced expression levels of SIRT1 and NAMPT in DEL as detected by transcriptome and confirmed by RQ-PCR analysis (Figure 5B, 5C). NAMPT activates deacetylase SIRT1 and deacetylation activates STAT3 via nuclear import [46, 65]. Since all analyzed ALCL cell lines expressed increased levels of NAMPT (Figure 5C), and high HLX expression significantly correlated with elevated NAMPT levels in ALCL patient samples (Figure 5D), these data highlighted its potential oncogenic function in this malignancy.

To identify downstream targets of HLX in ALCL we performed expression profiling of SU-DHL-1 cells treated for siRNA-mediated knockdown of HLX (Supplementary Table 6). RQ-PCR analysis of selected candidate target genes after HLX knockdown in DEL and SU-DHL-1 revealed that HLX activated JAK2, MSX1 and NKX2-2 and inhibited EOMES (Figure 5E). These results indicated that HLX performs auto-activation via JAK-STAT-signalling and deregulates lymphoid cell differentiation via specific developmental transcription factors.

Taken together, NKL homeobox gene HLX is aberrantly expressed in ALCL and activated by genomic copy number gain and transcription factor STAT3 which together represent an additional hallmark for ALCL. HLX was not expressed in normal ILC3/TH17 cells, disturbing the NKL-code if activated. HLX increases survival and deregulates cell differentiation, thus, representing a potent oncogene in this malignancy.

## DISCUSSION

**Figure 6:**
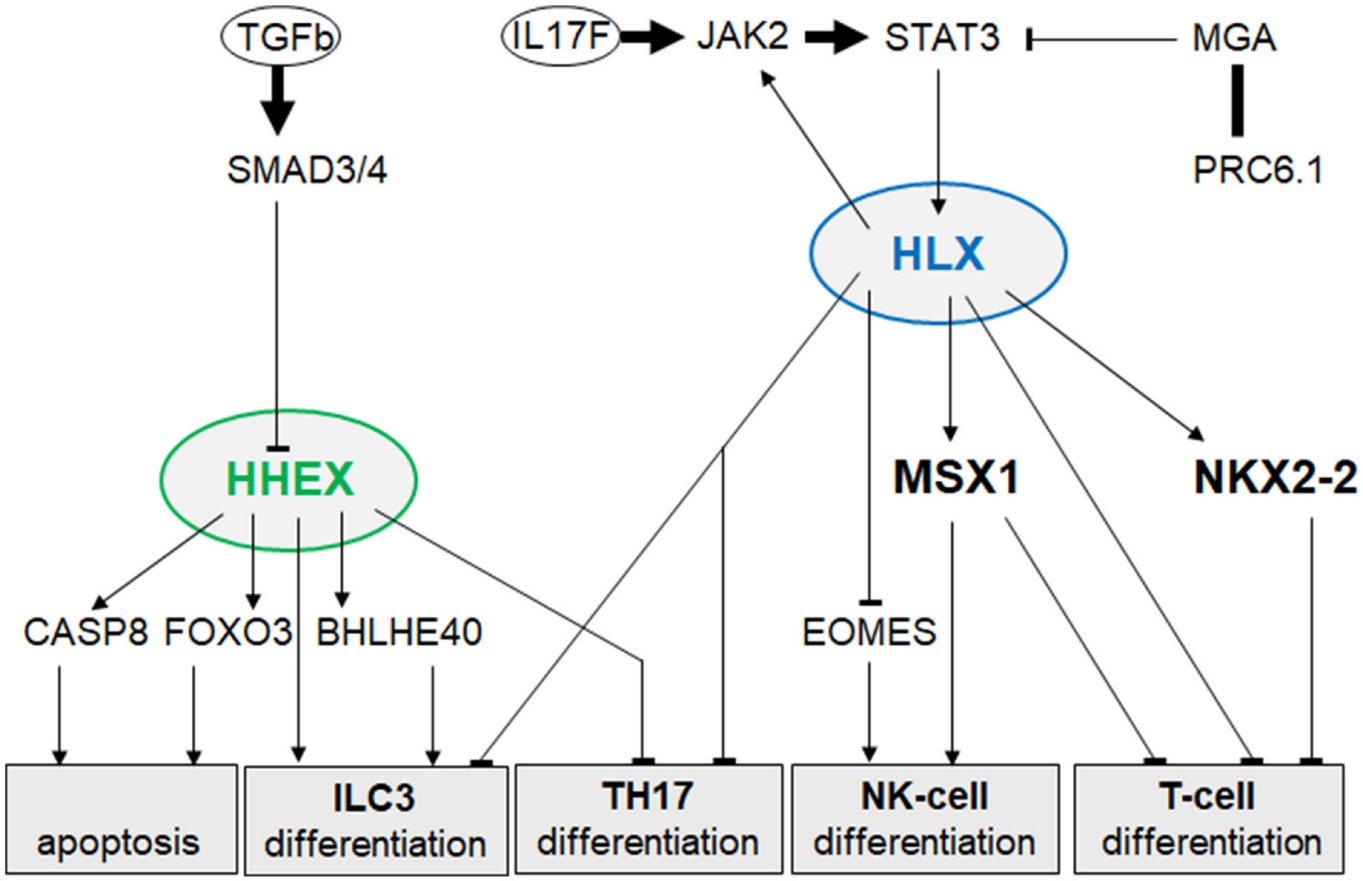
Gene regulatory network of HHEX and HLX in ALCL. This network summarizes identified regulators and target genes of NKL homeobox genes HHEX and HLX in ALCL. Their deregulated activity influences apoptosis and cell differentiation.

**Figure 7:**
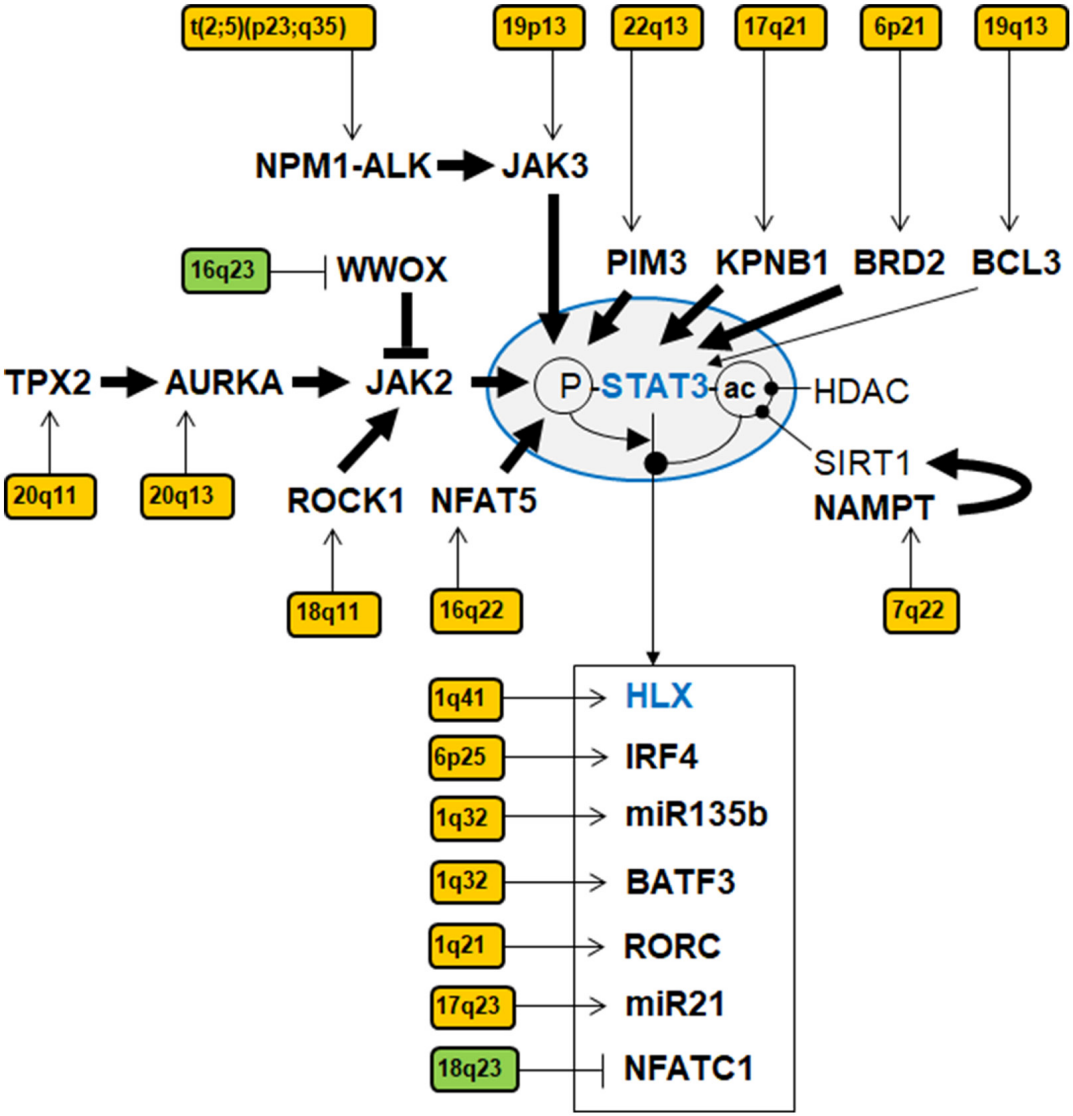
Gene regulatory network of STAT3 in ALCL. This network summarizes identified activators, inhibitors and target genes of STAT3 targeted by copy number alterations in ALCL cell lines. Copy number gains are indicated in yellow, copy number losses in green. Bold arrows indicate direct interactions. Phosphorylation (P) and acetylation (ac) of STAT3 are indicated as activating and inhibitory modifications, respectively.

Recently, we described the NKL-code which systematically delineates the expression patterns of eleven NKL homeobox genes in the hematopoietic system during early hematopoiesis, lymphopoiesis and myelopoiesis [13–15]. Here, we extend the NKL-code to cover the ILCs and identify expression of NKL homeobox gene HHEX exclusive to ILC3 at the expense of ILC1 and ILC2. This result was combined with our previous data resulting in a more complete NKL-code for the lymphoid system (Figure 1). ALCL cells express a TH17-like gene signature and - as recent literature suggests - are probably derived from ILC3 [28, 29]. Surprisingly, our study revealed reduced HHEX expression in ALCL cell lines reminiscent of normal TH17 cells in which HHEX is silenced. Supported by functional analyses, our data showed that HHEX operates as a tumor suppressor gene in ALCL, activating both apoptosis and differentiation (Figure 6). In contrast, NKL homeobox gene HLX was overexpressed in ALCL cell lines, supporting a dual role in promoting both cell survival and deregulation of differentiation genes (Figure 6). We demonstrated that STAT3 is a prominent and direct activator of HLX in ALCL. Our genomic data highlight copy number alterations in ALCL which target HLX, STAT3 and genes encoding STAT3-signalling factors (Figure 7). Thus, hallmark factor STAT3 deregulates HLX in ALCL, disturbing the respective NKL-code of ILC3 and TH17 cells.

HHEX serves as a master gene in hematopoiesis regulating major decisions in lineage differentiation [71]. Accordingly, HHEX is expressed in HSCs and progenitor cells. In lymphopoiesis HHEX remains active in memory B-cells and, as described here, in ILC3 [14]. Of note, ILC3-related TH17 cells failed to express HHEX. HHEX was inhibited in ALCL cells by the TGFbeta-pathway as reported in regulatory T-cells and prostate cells [37, 38]. SMAD3 is an operator of this pathway which inhibited HHEX expression and showed elevated expression levels in ALCL cell lines. SMAD7 performs inhibition of TGFbeta-signalling and is suppressed by miR21 [72]. This regulatory connection may also play a role in ALCL, since miR21/VMP1 was elevated in ALCL cell lines, is overexpressed in several T-cell lymphomas, and has been shown to support TH17 differentiation [73–75]. Furthermore, TGFbeta-signalling drives TH17 differentiation [76], so this pathway may also contribute to the physiological downregulation of HHEX expression in TH17 cells.

Functional analyses indicated that HHEX activates the p53-pathway in addition to CASP8 and FOXO3 which are both regulatorily connected and drivers of apoptosis [77, 78]. In contrast, NPM1-ALK suppresses FOXO3, highlighting the necessity of its abatement and that of apoptosis for ALCL [79]. BHLHE40 expression was found to be elevated in ILC3 and activated by HHEX. BHLHE40 is also activated by IL1-signalling – a key pathway in ILC3 [80]. In addition, BHLHE40 is involved in B-cell differentiation and its aberrant downregulation may play a role in HL [81, 82]. These findings functionally connect HHEX with differentiation processes in ILC3. Collectively, HHEX was downregulated and impacted apoptosis and differentiation thus performing tumor suppressor gene activities both in ILC3 and ALCL (Figure 6). However, in subsets of both, ALCL and T-ALL HHEX operates as an oncogene [13, 35]. Thus, HHEX is the second identified NKL homeobox gene after MSX1 representing a tumor suppressor as well as an oncogene [17, 18]. All ALCL cell lines analyzed in this study showed tumor suppressor activity for HHEX. Therefore, we were unable to examine the oncogenic role of HHEX indicated for subsets of ALCL patients in a cell line model.

HLX is firmly expressed in normal hematopoiesis and belongs to the NKL-code as well as HHEX [13, 83]. HLX operates as a fundamental regulator of hematopoietic and lymphopoietic processes [11]. Thus, both HHEX and HLX share these basic developmental functions in this tissue. However, in contrast to HHEX which remains active in memory B-cells and ILC3, HLX is silenced in all final stages of lymphoid differentiation including those in ILC3 and TH17 cells [13, 14]. This observation might indicate that HLX possesses particular oncogenic potency if aberrantly expressed in lymphoid cells. In accord with this notion, our results also showed that HLX activated NKX2-2. This NKL homeobox gene while not included in the NKL-code operates as an oncogene in T-ALL and HL [84, 85], reinforcing its impact on the deregulation of lymphoid differentiation, an oncogenic activity which may also play a role in ALCL. HLX also deregulates apoptosis and B-cell differentiation and is aberrantly expressed in HL and EBV-positive DLBCL [14, 86]. Here, we identified aberrant activity of HLX in ALCL, indicating deregulation of apoptosis and of processes mediating ILC, T-cell and NK-cell differentiation (Figure 6).

STAT3 serves as a major oncogene in ALCL where it is aberrantly activated by fusion gene NPM1-ALK via JAK2 and JAK3 or by particular mutations [22, 23]. The ALCL cell lines used in this study all bore the fusion gene NPM1-ALK. Consistently, LL-100 transcriptome data indicated absence of mutations in STAT3. Our genomic profiling data revealed 17 deregulated genes functionally connected with STAT3, including KPNB1 and NAMPT. KPNB1 encodes karyophorin subunit beta 1 which belongs to the importin family, specifically performing nuclear import of proteins. Accordingly, KPNB1 mediates the transfer of STAT3 (and NKX2-2) into the nucleus [56, 87]. Thus, enhanced expression of KPNB1 may result in increased transcriptional activity of STAT3. NAMPT encodes the enzyme nicotinamide phosphoribosyltransferase which interacts with and activates SIRT1 [46]. SIRT1 possesses deacetylase activity and has been shown to regulate STAT3 [88]. However, our results indicated an activatory role of NAMPT/SIRT1 for STAT3 while Limagne and coworkers have shown that deacetylated STAT3 is inactivated [88]. Of note, particular sites of acetylation may affect STAT3 differently, explaining possible contradictions. Nevertheless, SIRT1 is also a target gene of STAT3 in TH17 cells [89], underlining the regulatory connection between these genes. Thus, in HL and ALCL SIRT1 probably coactivates STAT3 via deacetylation-mediated nuclear import [65]. Together, the upregulated genes KPNB1, NAMPT and SIRT1 in ALCL cell lines highlight the role of the (de) regulation of subcellular localization of STAT3 in lymphomagenesis.

ALCL cells express several TH17 signature genes and share similarities with ILC3 from which these malignant cells may derive [8, 28, 29]. Genes targeted by copy number gain in ALCL cell lines included BATF3, IRF4, STAT3 and RORC. They belong to the TH17 gene signature [90], supporting the indicated character of ALCL. Breast implant-associated (BIA)-ALCL, CTLC and ALCL are closely related malignancies and consistently express several aspects of ILC3/TH17 cells aberrantly [28, 69, 91]. In CTCL, IL17F expression is driven via the IL2-JAK3-STAT5 pathway reportedly activated by a particular mutation of SOCS1 [69]. However, our data revealed absence of SOCS1 mutations in ALCL cell lines, excluding this mode of IL17F activation in ALCL. AP1 factor BATF has been shown to activate IL17F directly and JUNB supports TH17 differentiation, highlighting the connection between AP1, TH17 and ALCL [92, 93]. Furthermore, IL17F expression has been detected in ILC3 cells of the skin [94], revealing a functional role for this cytokine in this type of innate immune cells. However, since BATF3 was highly expressed in all ALCL cell lines analyzed, this factor may not represent the major driver of IL17F exclusively in SUP-M2. Of note, HHEX interacts and thereby inhibits the activity of AP1 factors [95]. This feature of HHEX may contribute to its potential roles in suppression of TH17 cell differentiation and in tumor suppressor function in ALCL.

Our study of NKL homeobox genes HHEX and HLX in ALCL documents their impact in (deregulated) cell differentiation: HLX as oncogene and HHEX as tumor suppressor gene. Our data on NKL homeobox gene activities support the reported close relation between normal ILC3/TH17 cells and ALCL. ILCs and TH-cells show high degrees of plasticity [96]. This feature may result from these close relationships between normal ILC3 and TH17 cells and may underlie the different characteristics of the derived T-cell lymphoma subtypes. Developmental plasticity may offer a starting-point for novel rational therapies such as that using retinoids proven effective against acute promyelocytic leukemia to drive the tumor into differentiation along related non-malignant pathways. ALCL cell lines analyzed in this study are well characterized tumor models and may serve as *in vitro* tools to advance this strategy.

## MATERIALS AND METHODS

### Transcriptome analysis, expression profiling and bioinformatic analyses

Transcriptome data from primary ILCs were obtained from Gene Expression Omnibus (GEO; https://www.ncbi.nlm.nih.gov/gds) using datasets GSE112591, GSE124474 and GSE90834 and obtained from ArrayExpress (AE; https://www.ebi.ac.uk/) using dataset E-MTAB-8494 [30–33]. Expression values for each ILC type were averaged and listed in Supplementary Tables 1–4. Transcriptome data of primary TH17 cells were obtained from dataset GSE107011, using the associated online tool ABIS [34]. Transcriptome data from 100 leukemia/lymphoma cell lines (LL-100) were obtained from the European Nucleotide Archive (ENA; https://www.ebi.ac.uk/ena) using dataset PRJEB30312 [97]. Graphical presentations of the LL-100 data and the generation of a dendrogram via hierarchical clustering by the Ward‘s method were performed using shinyNGS (https://github.com/pinin4fjords/shinyngs). Chromatin immuno-precipitation (ChIP)-sequencing (seq) data for STAT3 in ALCL cell line SU-DHL-1 were from GEO-dataset GSE117164 [66]. ChIP-seq data for MGA in 293 cells were from ENA-dataset E-MTAB-6006 [70]. All ChIP-seq data were analyzed using the Integrative Genomics Viewer (obtained from the Broad Institute, https://www.broadinstitute.org/data-software-and-tools).

Expression profiling datasets of T-cell lymphoma patients were obtained from GEO and used to examine ALCL (GSE19069 and GSE14879) and peripheral T-cell lymphoma (GSE6338) patients [23, 28, 86]. Data were analyzed using the associated online tool GEO2R. Expression profiling datasets from treated ALCL cell line SU-DHL-1 were generated by Dr. Robert Geffers (Genome Analytics, Helmholtz Centre for Infection Research, Braunschweig, Germany) using HG U133 Plus 2.0 gene chips (Affymetrix, High Wycombe, UK). The primary data are available at GEO via GSE146391. After RMA-background correction and quantile normalization of the spot intensities, the profiling data were expressed as ratios of the sample mean and subsequently log2 transformed. Data processing was performed via R/Bioconductor using limma and affy packages. To parse biological function of 1000 shortlisted genes, gene-annotation enrichment analysis was performed using DAVID bioinformatics resources (https://david.ncifcrf.gov/) [98].

### Cell lines and treatments

ALCL-derived cell lines (DEL, KI-JK, L-82, SR-786, SU-DHL-1, SUP-M2) in addition to HL-derived cell line L-540 and DLBCL-derived cell line DOHH-2. All cell lines have been obtained from DSMZ (German Collection of Microorganisms and Cell Lines - Deutsche Sammlung von Mikroorganismen und Zelllinien, Braunschweig, Germany), a public, non-profit biological ressources center owned by the German government. Cell culture conditions, culture media and other relevant information on each cell line are provided in detail on the institute`s website at https://www.dsmz.de/ [41, 99]. This cell line panel is monitored and validated by a unique program of intensity and quality which is rigorously implemented for all cell lines like authentication, exclusion of cross-contamination, documentation of freedom from inadvertent mycoplasm and viral contamination [100, 101].

Cell stimulations were performed for 16 h by treatment with 20 ng/ml recombinant human protein TGFbeta (240-B, R&D Systems, Wiesbaden, Germany), inhibitory antibody directed against IL17F (8134-IL-025/CF, R&D Systems), 10 μg/ml trichostatin A (TSA, T8552, Sigma, Taufkirchen, Germany), 50 μM resveratrol (R5010, Sigma), 100 μM AG490 (T3434, Sigma), or 1 μM crizotinib (PZ0240, Sigma).

Gene specific siRNA oligonucleotides and AllStars negative Control siRNA (siCTR) were purchased from Qiagen (Hilden, Germany). Expression constructs for HHEX were purchased from Origene (Wiesbaden, Germany). SiRNAs (80 pmol) and expression constructs/vector controls (2 μg) were transfected into 1 × 10^6^ cells by electroporation using the EPI-2500 impulse generator (Fischer, Heidelberg, Germany) at 350 V for 10 ms. Transfected cells were harvested after 20 h cultivation. All stimulation experiments were performed twice generating similar results.

For functional testing treated cells were analyzed with the IncuCyte S3 Live-Cell Analysis System (Essen Bioscience, Hertfordshire, UK). For detection of apoptotic cells we additionally used the IncuCyte Caspase-3/7 Green Apoptosis Assay diluted at 1:2000 (Essen Bioscience). Live-cell imaging experiments were performed twice with fourfold parallel tests.

### Chromosomal and genomic analyses

Karyotyping and chromosomal analysis by fluorescence *in situ* hybridization (FISH) was performed as described previously [102]. BAC clones were obtained from BacPac Resources, Children´s Hospital Oakland Research Institute (CA, USA) to analyze IL17F at 6p12 (RP11-156F16, RP11-318H5 and RP11-367A5). According to genome release hg19, the sequences of the BAC clones correspond to positions 50.0 Mb, 52.1 Mb, and 79.4 Mb, respectively. Insert DNA was harvested using the Big BAC DNA Kit (Princeton Separations, Adelphia, NJ, USA) and directly labelled by nick translation with dUTP-fluors (Dyomics, Jena, Germany). Fluorescent images were captured and analyzed with an Axio-Imager microscope (Zeiss, Göttingen, Germany) configured to a dual Spectral Imaging FISH system (Applied Spectral Imaging, Vätö, Sweden).

For genomic profiling genomic DNA of ALCL cell lines was prepared by the Qiagen Gentra Puregene Kit (Qiagen). Labelling, hybridization and scanning of HD Cytoscan arrays was performed at the Genome Analytics Facility, Helmholtz Centre for Infection Research (Braunschweig, Germany), using HD arrays according to the manufacturer´s protocols (Affymetrix). Data were visualized and interpreted using the Chromosome Analysis Suite software version 2.0.1.2 (Affymetrix).

### Polymerase chain-reaction (PCR) analyses

Total RNA was extracted from cell line samples using TRIzol reagent (Invitrogen, Darmstadt, Germany). cDNA was synthesized by random priming from 5 μg RNA using Superscript II (Invitrogen). Real-time quantitative (RQ)-PCR analysis was performed with the 7500 Real-time System, using commercial buffer and primer sets (Thermo Fisher Scientific, Darmstadt, Germany). Quantification of MSX1 was performed as described previously [35]. For normalization of expression levels we analyzed the transcript of TATA box binding protein (TBP). We used the ddCT-method and the obtained values are indicated as fold expression in relation to one selected sample which was set to 1.

For quantification of HLX copy numbers we used the following oligonucleotides: HLX-1 5′-GGAAGCTTGTGCCGCTCTCCCGGGTTTCG-3′ and HLX-2 5′-TTGGATCCAGGAGTCCGTGTCCTCGGCAAAGC-3′. The locus of MSX1 was used as control: MSX1-1 5′-GTGGACTCCAGGTGCCCAAG-3′ and MSX1-2 5′-CGGCCATTAGGCTTAGGGAG-3′. Oligonucleotides were obtained from Eurofins MWG (Ebersberg, Germany). Genomic DNA was prepared as described above.

Quantitative PCR analyses were performed in triplicate. Standard deviations are calculated for each experiment and presented in the figures as error bars. Statistical significance was assessed by *t*-Test and derived *p*-values indicated by asterisks (^*^ *p* < 0.05, ^**^ *p* < 0.01, ^***^ *p* < 0.001, n. s. not significant).

For detection of NPM1-ALK fusion transcripts and NPM1 controls we performed reverse transcription (RT)-PCR, using the following oligonucleotides: NPM1-for 5′-CGATGGACATGGACATGAGC-3′, NPM1-rev 5′-ACTTCAACCTTAAGACCACTGG-3′, and ALK-rev 5′-ATCTGCATGGCTTGCAGCTC-3′, all purchased from Eurofins MWG. PCR products were generated using taqpol (Qiagen) and thermocycler TGradient (Biometra, Göttingen, Germany), analyzed by gel electrophoresis and documented with the Azure c200 Gel Imaging System (Azure Biosystems, Dublin, CA, USA).

### Protein analyses

Western blots were generated by the semi-dry method. Protein lysates from cell lines were prepared using SIGMAFast protease inhibitor cocktail (Sigma). Proteins were transferred onto nitrocellulose membranes (Bio-Rad, München, Germany) and blocked with 5% dry milk powder dissolved in phosphate-buffered-saline buffer (PBS). The following antibodies were used: alpha-Tubulin (T6199, Sigma), HLX (Novus Biologicals, Abingdon, UK), STAT3 (9139, Cell Signalling, Leiden, Netherlands) and phospho-STAT3 (9136, Cell Signalling). For loading controls blots were reversibly stained with Poinceau (Sigma) and detection of alpha-Tubulin (TUBA) was performed thereafter. Secondary antibodies were linked to peroxidase for detection by Western-Lightning-ECL (Perkin Elmer, Waltham, MA, USA). Documentation was performed using the digital system ChemoStar Imager (INTAS, Göttingen, Germany).

IL17F protein was quantified in cell culture supernatants using the IL-17 Human ELISA kit (Thermo Fisher Scientific). To harvest the supernatants 1 × 10^6^ cells were washed in PBS and cultured in 1 ml fresh medium. After 24 hours supernatants were centrifuged and stored at –20°C. To optimize the range of the kit supernatants were diluted 1:50 and 1:100 in PBS.

## SUPPLEMENTARY MATERIALS














