# Supplementary material for: The NKL-code for innate lymphoid cells reveals deregulated expression of NKL homeobox genes HHEX and HLX in anaplastic large cell lymphoma (ALCL)
Source: Oncotarget. 2020 Aug 25;11(34):3208–26. doi: 10.18632/oncotarget.27683 (PMC7456612; doi:10.18632/oncotarget.27683)
Supplement: Supplementary file 1 [file oncotarget-11-3208-s001.pdf]

The NKL-code for innate lymphoid cells reveals deregulated expression of NKL homeobox genes HHEX and HLX in anaplastic large cell lymphoma (ALCL)

SUPPLEMENTARY MATERIALS

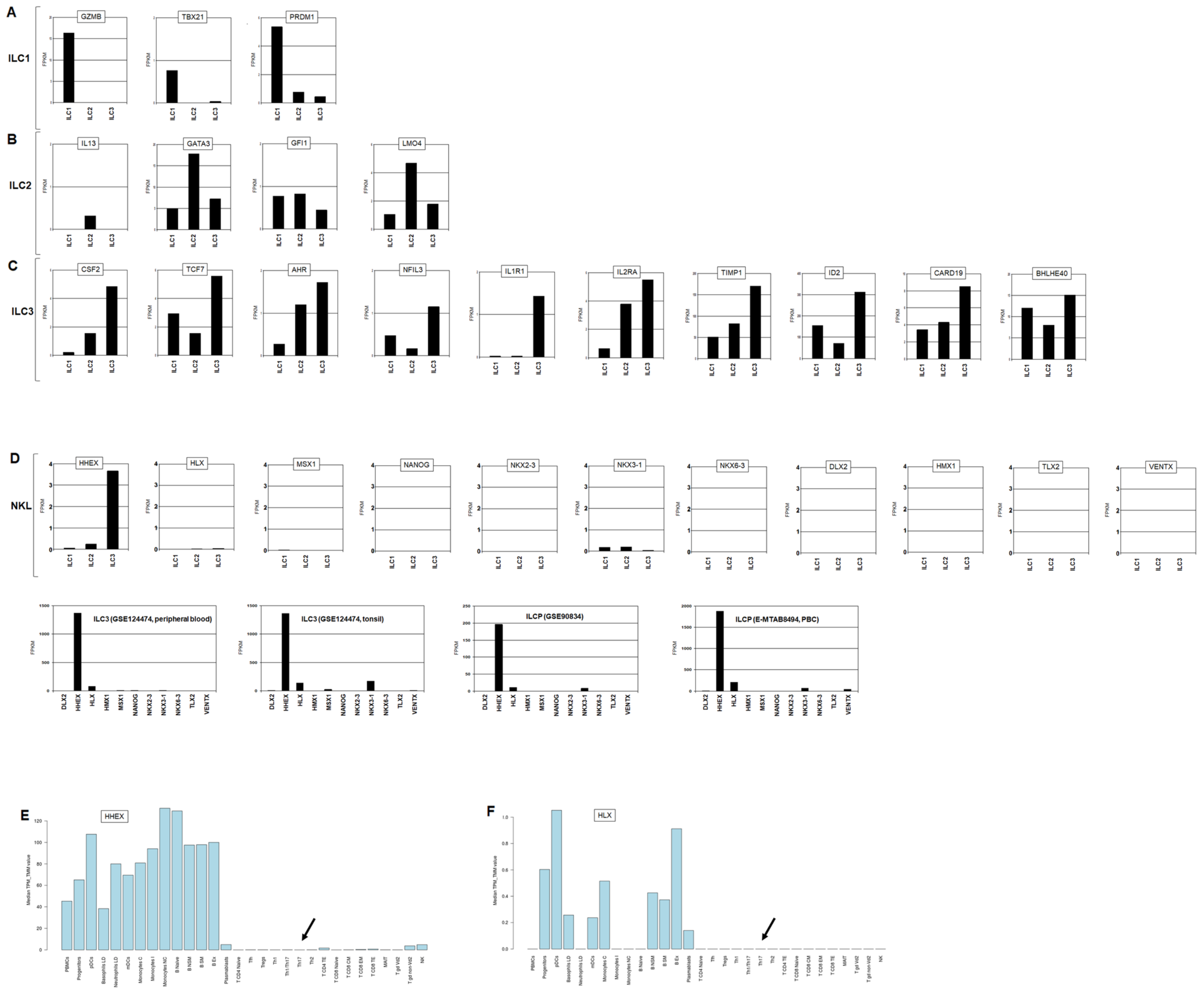

Supplementary Figure 1: Gene expression analysis in ILCs and various immune cells. Differential gene activities in (**A**) ILC1, (**B**) ILC2, (**C**) ILC3, and of (**D**) NKL code members according to dataset GSE112591 (above) and datasets GSE124474, GSE90834 and E-MTAB-8494 (below). Expression levels of (**E**) HHEX and (**F**) HLX in several types of immune cells according to dataset GSE107011. Note absent expression in TH17 cells (indicated by arrows) of both HHEX and HLX.

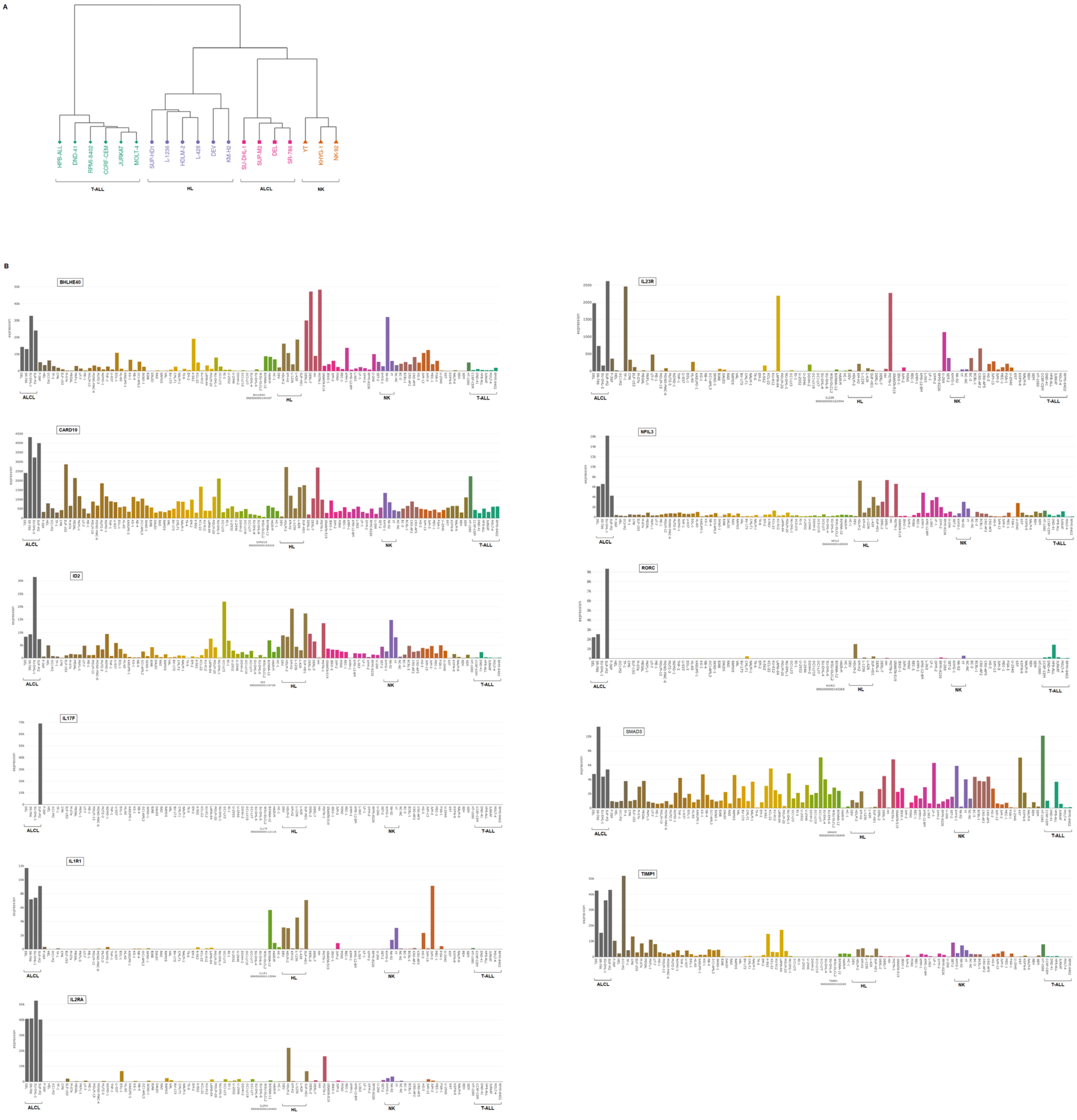

Supplementary Figure 2: LL-100 transcriptome data (dataset PRJEB30312). (**A**) Clustering of cell lines according to their derived malignancies from T-ALL, HL, ALCL and NK-cell. (**B**) Expression levels of ILC3/TH17 signature genes BHLHE40, CARD10, ID2, IL17F, IL1R1, IL2RA, IL23R, NFIL3, RORC, TIMP1 in addition to TGFbeta-pathway operator SMAD3.

A

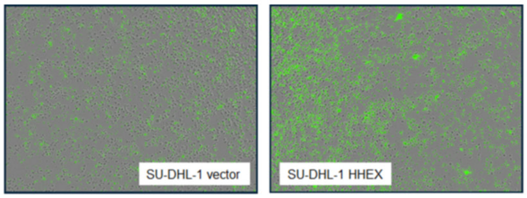

18h, apoptotic cells are indicated in green

B **SUDHL1-HHEX, upregulated (top-1000)**

Sublist	Category	Term	RT	Genes	Count	%	P-Value	Benjamini
	KEGG_PATHWAY	Circadian entrainment	RT		10	1,5	8,6E-4	1,8E-1
	KEGG_PATHWAY	Dopaminergic synapse	RT		11	1,7	2,0E-3	2,0E-1
	KEGG_PATHWAY	Serotonergic synapse	RT		10	1,5	2,6E-3	1,8E-1
	KEGG_PATHWAY	Calcium signaling pathway	RT		13	2,0	2,7E-3	1,4E-1
	KEGG_PATHWAY	Pathways in cancer	RT		20	3,0	7,7E-3	2,9E-1
	KEGG_PATHWAY	Cholinergic synapse	RT		9	1,4	8,9E-3	2,8E-1
	KEGG_PATHWAY	PI3K-Akt signaling pathway	RT		18	2,7	9,6E-3	2,7E-1
	KEGG_PATHWAY	Retrograde endocannabinoid signaling	RT		8	1,2	1,7E-2	3,9E-1
	KEGG_PATHWAY	Salivary secretion	RT		7	1,1	2,6E-2	4,8E-1
	KEGG_PATHWAY	Adrenergic signaling in cardiomyocytes	RT		9	1,4	3,0E-2	4,9E-1
	KEGG_PATHWAY	Glutamatergic synapse	RT		8	1,2	3,1E-2	4,7E-1
	KEGG_PATHWAY	p53 signaling pathway	RT		6	0,9	3,2E-2	4,5E-1
	KEGG_PATHWAY	Morphine addiction	RT		7	1,1	3,3E-2	4,4E-1
	KEGG_PATHWAY	Intestinal immune network for IgA production	RT		5	0,8	3,5E-2	4,3E-1
	KEGG_PATHWAY	Ras signaling pathway	RT		12	1,8	3,7E-2	4,3E-1
	KEGG_PATHWAY	Gastric acid secretion	RT		6	0,9	4,3E-2	4,6E-1
	KEGG_PATHWAY	Oxytocin signaling pathway	RT		9	1,4	4,5E-2	4,6E-1
	KEGG_PATHWAY	Autoimmune thyroid disease	RT		5	0,8	4,8E-2	4,6E-1
	KEGG_PATHWAY	Rap1 signaling pathway	RT		11	1,7	5,2E-2	4,7E-1
	KEGG_PATHWAY	Hypertrophic cardiomyopathy (HCM)	RT		6	0,9	5,5E-2	4,7E-1
	KEGG_PATHWAY	Non-small cell lung cancer	RT		5	0,8	6,0E-2	4,8E-1
	KEGG_PATHWAY	Viral myocarditis	RT		5	0,8	6,3E-2	4,9E-1
	KEGG_PATHWAY	MAPK signaling pathway	RT		12	1,8	7,2E-2	5,2E-1
	KEGG_PATHWAY	Allograft rejection	RT		4	0,6	7,3E-2	5,1E-1
	KEGG_PATHWAY	Cell adhesion molecules (CAMs)	RT		8	1,2	8,2E-2	5,4E-1
	KEGG_PATHWAY	Synaptic vesicle cycle	RT		5	0,8	8,5E-2	5,3E-1
	KEGG_PATHWAY	Tuberculosis	RT		9	1,4	9,7E-2	5,7E-1

SUDHL1-HHEX, downregulated (top-1000)

Sublist	Category	Term	RT	Genes	Count	%	P-Value	Benjamini
	KEGG_PATHWAY	T cell receptor signaling pathway	RT		10	1,4	6,9E-3	7,9E-1
	KEGG_PATHWAY	Focal adhesion	RT		14	1,9	2,4E-2	9,3E-1
	KEGG_PATHWAY	Hippo signaling pathway	RT		11	1,5	3,4E-2	9,2E-1
	KEGG_PATHWAY	Proteoglycans in cancer	RT		13	1,8	4,1E-2	9,0E-1
	KEGG_PATHWAY	Cell adhesion molecules (CAMs)	RT		10	1,4	5,4E-2	9,2E-1
	KEGG_PATHWAY	Natural killer cell mediated cytotoxicity	RT		9	1,2	5,7E-2	8,9E-1
	KEGG_PATHWAY	TGF-beta signaling pathway	RT		7	0,9	6,7E-2	8,9E-1
	KEGG_PATHWAY	Nicotinate and nicotinamide metabolism	RT		4	0,5	7,5E-2	8,9E-1
	KEGG_PATHWAY	NF-kappa B signaling pathway	RT		7	0,9	7,6E-2	8,6E-1
	KEGG_PATHWAY	Arrhythmogenic right ventricular cardiomyopathy (ARVC)	RT		6	0,8	7,8E-2	8,4E-1
	KEGG_PATHWAY	Calcium signaling pathway	RT		11	1,5	8,6E-2	8,4E-1
	KEGG_PATHWAY	Neuroactive ligand-receptor interaction	RT		15	2,0	9,1E-2	8,3E-1
	KEGG_PATHWAY	MAPK signaling pathway	RT		14	1,9	9,2E-2	8,1E-1
	KEGG_PATHWAY	Pancreatic secretion	RT		7	0,9	9,8E-2	8,1E-1
	KEGG_PATHWAY	Leukocyte transendothelial migration	RT		8	1,1	9,8E-2	7,9E-1

Supplementary Figure 3: Functional analyses of HHEX in ALCL cells. (**A**) Live-cell imaging pictures of SU-DHL-1 treated for overexpression of HHEX after 18 hours. Apoptotic cells are indicated in green. (**B**) The top-1000 differentially expressed genes were obtained by expression profiling after forced HHEX expression in SU-DHL-1 and analyzed using DAVID. Arrows indicate upregulated p53-signalling pathway (left) and inhibited NFkB-pathway (right) by HHEX.

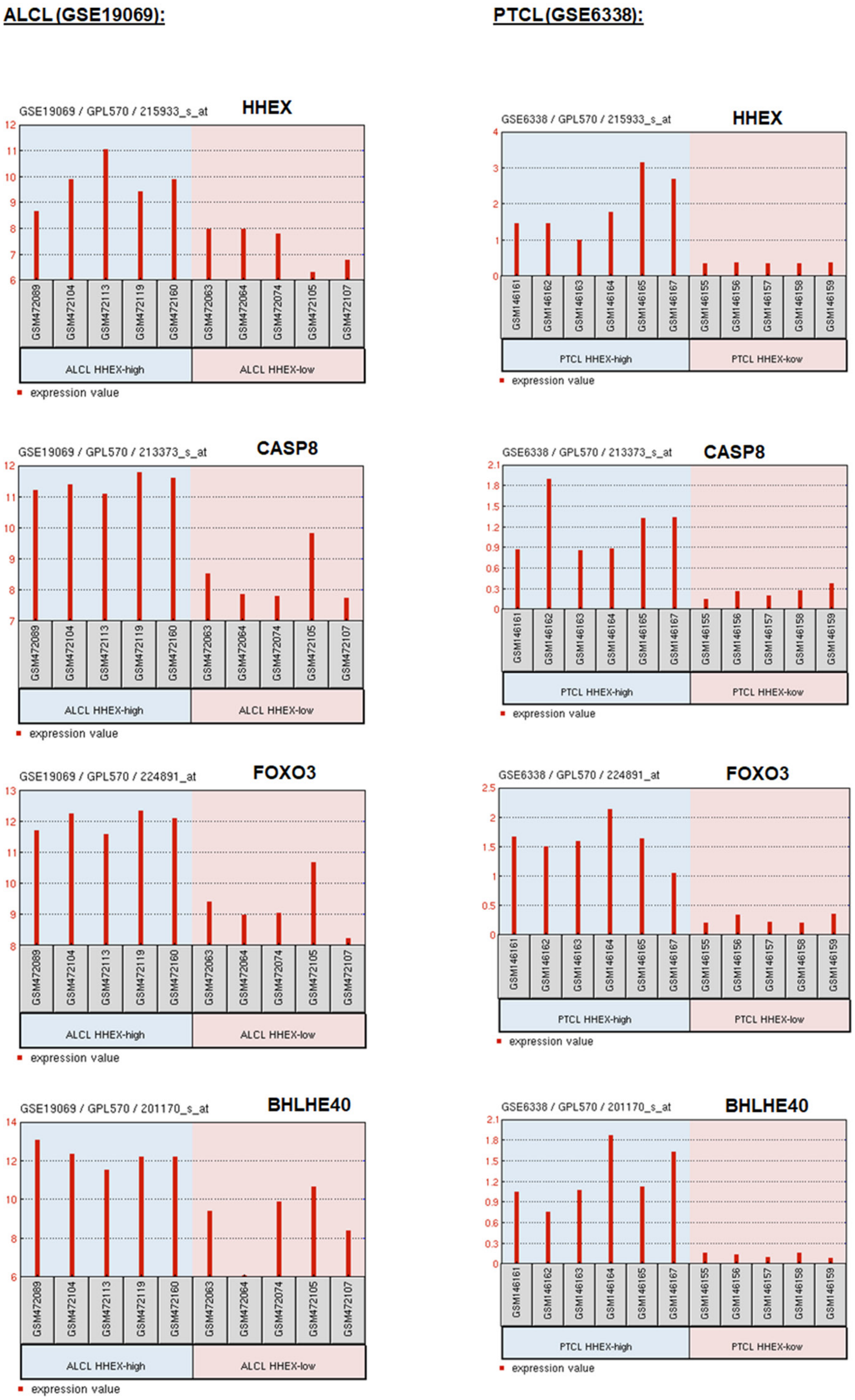

Supplementary Figure 4: Correlated gene expressions of HHEX in ALCL and PTCL. Selected ALCL and PTCL patients expressing high and low levels of HHEX were analyzed using GEO online tool GEOR. The obtained data indicate correlated expression of HHEX, CASP8, FOXO3 and BHLHE40 in ALCL (dataset GSE19069, on the left) and PTCL (dataset GSE6338, on the right) patients.

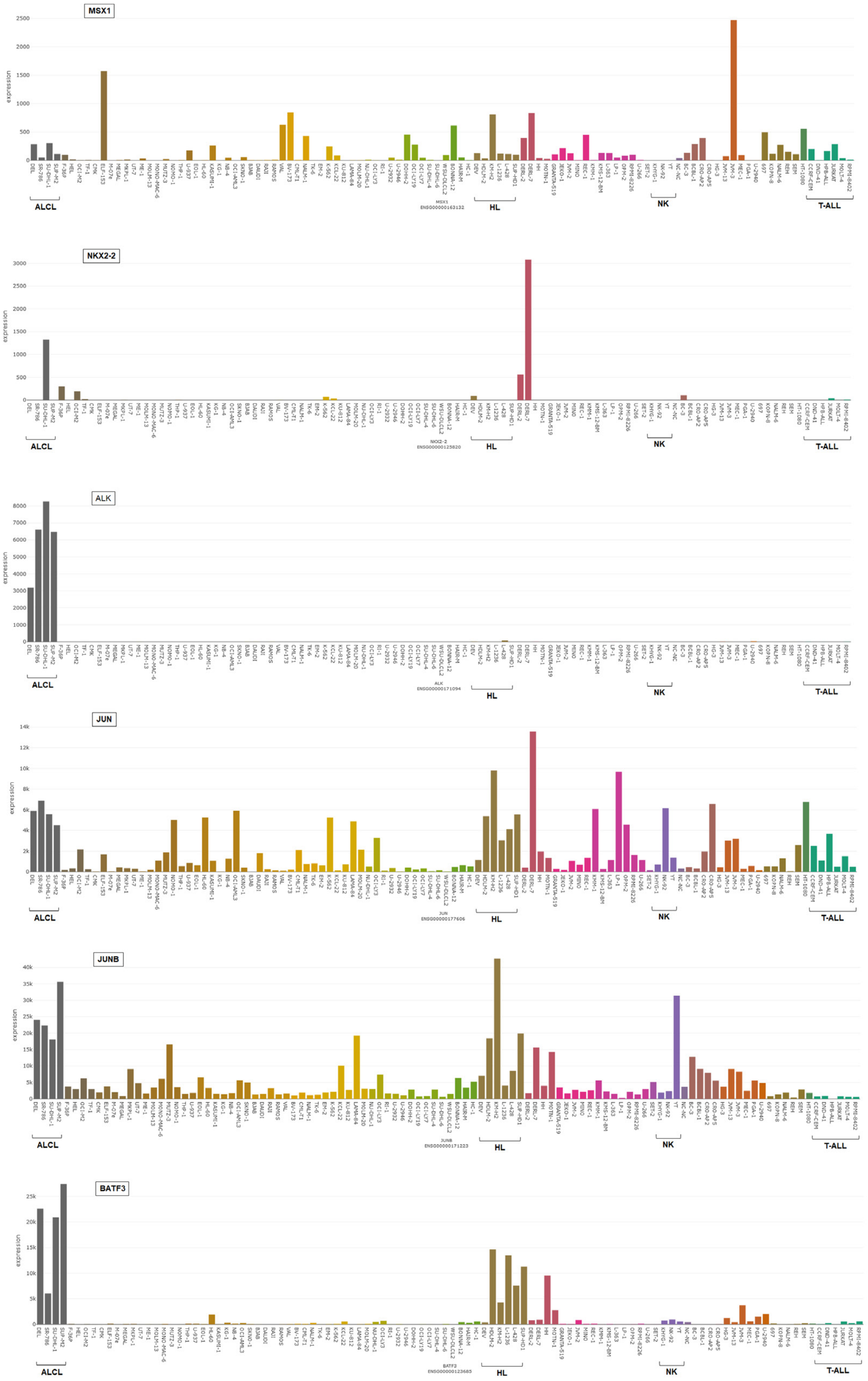

Supplementary Figure 5: LL-100 transcriptome data. For NKL homeobox genes MSX1 and NKX2-2, for ALK, and for AP1 factors JUN, JUNB and BATF3.

A

DEL:
47(44-49)<2n>XY,+5,+5,der(5)t(5;6)(q35;p21), t(5;6)(q35;p21),+6,-8,add(10)(q23-24),der(13)t(1;13)(q32;p11)t(1;13)(q21;q34), add(16)(q23),add(19)(p13)+mar.

L-82:
72-77<3n>XX/XXX,add(X)(q12),add(X)(q27),+1,add(1)(p11),+2,t(2;5)(p23;q35),der(2)t(2;5)(p23;q35),der(4)add4(p14q35),+5, der(6)i(6)(q10)t(1;6)(q41;p24),+7,add(7)(q22),add(8)(q24),+9,add(9)(p2?2)x2,add(9)(q11),+10,add(10)(p1?),add(13)(q33), der(13)t(13;15)(p11;q14),-14,-15,del(16)(q23),add(17)(p1?),+4-6mar.

SR-786: 70-84<3n>XX?Y,add(1q11),del(1)(p11)/der(?)t(1;?)(q11;?),+2,der(2)t(2,5)(p23;q35)inv(2)(p23q14)x2,-4,del(4)(q22),+5, der(5)t(2;5)(p23;q35)x2,+6,+7,del(7)(q21),+8,der(9)t(1;9)(q11;p24)x2,der(12)t(12;13)(q24.32;q11)x1-2,-13,-13,del(13)(q13q31), +14,add(14)(p11)/der(?)t(14;?)(q11;?)x2,-18,+19,del(21)(q22),+22,+6-9mar.

SU-DHL-1:
74(67-75)<3n>XX,-Y,1,del(1)(p21),+2,t(2;5)(p23;q35)x2,+3,+5,del(6)(q23)x1-2,-7,add(8)(p12),add(9)(p21),del(10)(p14),+12, add(12)(q24),add(14)(p12),-16,add(16)(q24),-18,+19,dup(19)(q13.1qter),-20,+21,+3mar.

SUP-M2:
44-50<2n>XX/XXX,+1,add(X)(q26),der(X;7)(q10;p10),add(1)(q21),der(1)(1pter→q43::1q43→q21::9q31→qter),t(2;5)(p23;q35), der(6)t(6;6)(q27;p11),+7,+8,add(9)(q31),add(11)(q24)/der(11)t(4;11)(?q34;q24), del(19)(q13.2).

B

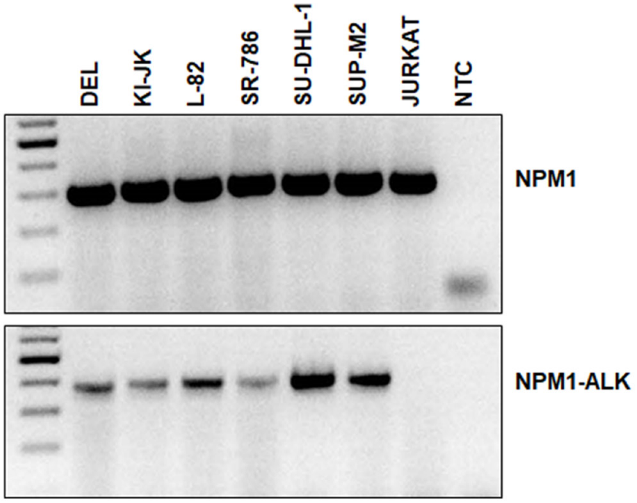

Supplementary Figure 6: Karyotyping of ALCL cell lines. (**A**) Karyotypes of ALCL cell lines DEL, L-82, SR-786, SU-DHL-1 and SUP-M2. (**B**) RT-PCR analysis of control NPM1 and fusion gene NPM1-ALK in ALCL cell lines. JURKAT served as control, NTC: no template control.

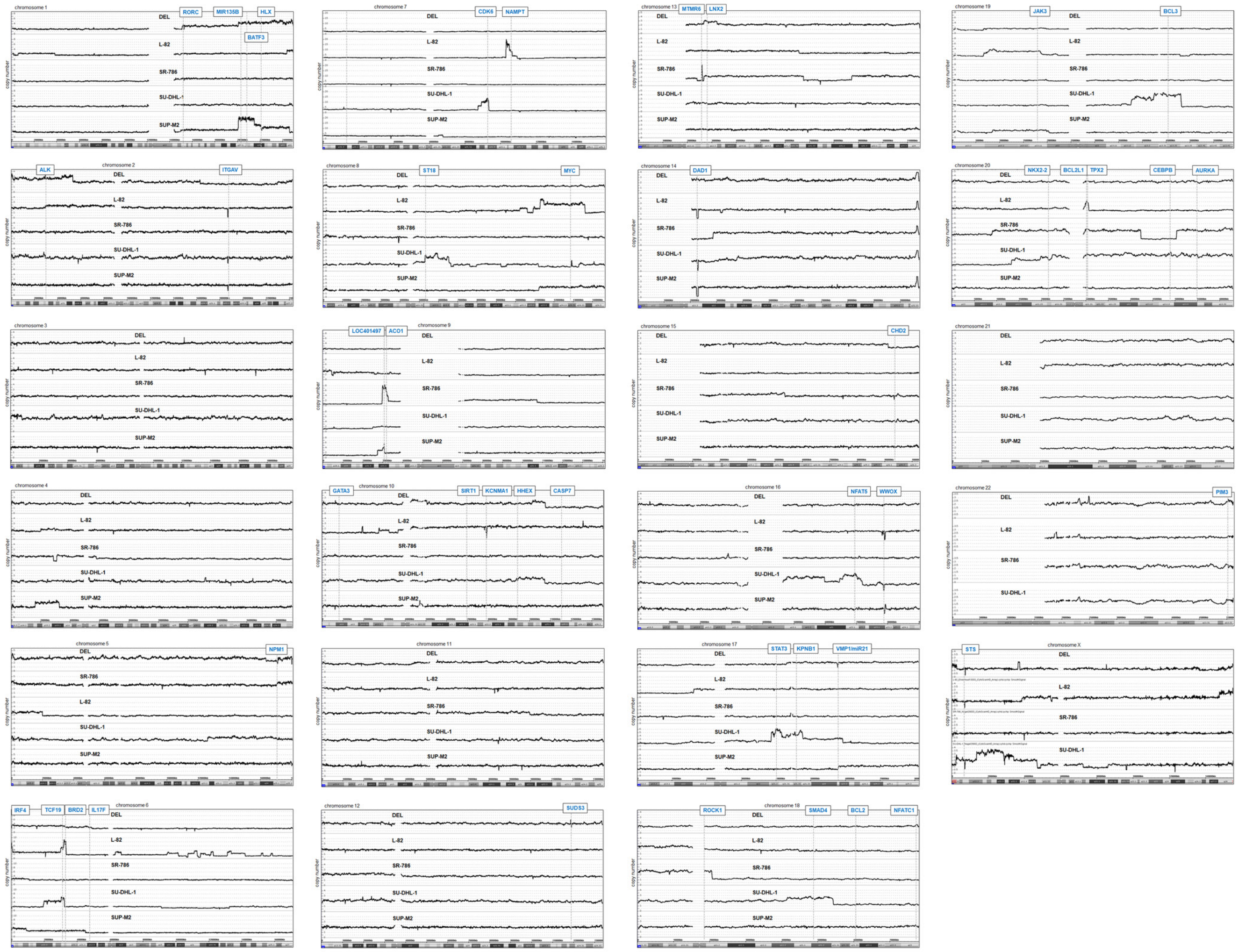

Supplementary Figure 7: Copy number analysis of ALCL cell lines. Copy number alterations and selected gene targets are indicated for ALCL cell lines DEL, L-82, SR-786, SU-DHL-1 and SUP-M2.

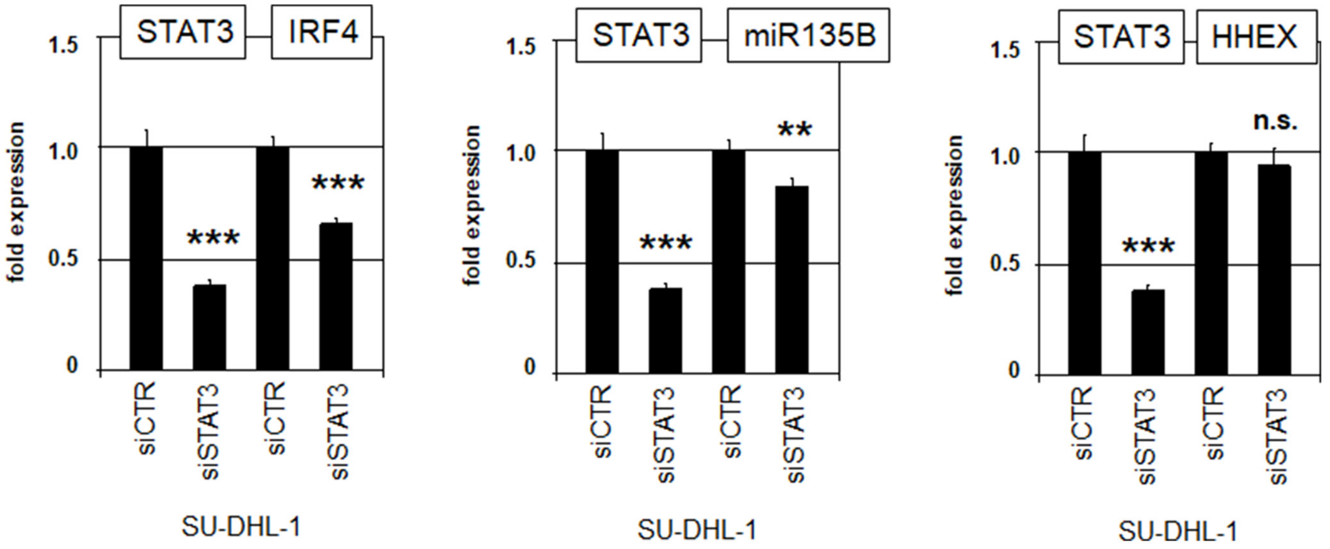

Supplementary Figure 8: STAT3 target gene analysis. RQ-PCR of STAT3 reported target genes IRF4 (left) and miR135B (middle), and of HHEX (right) after siRNA-mediated knockdown of STAT3.

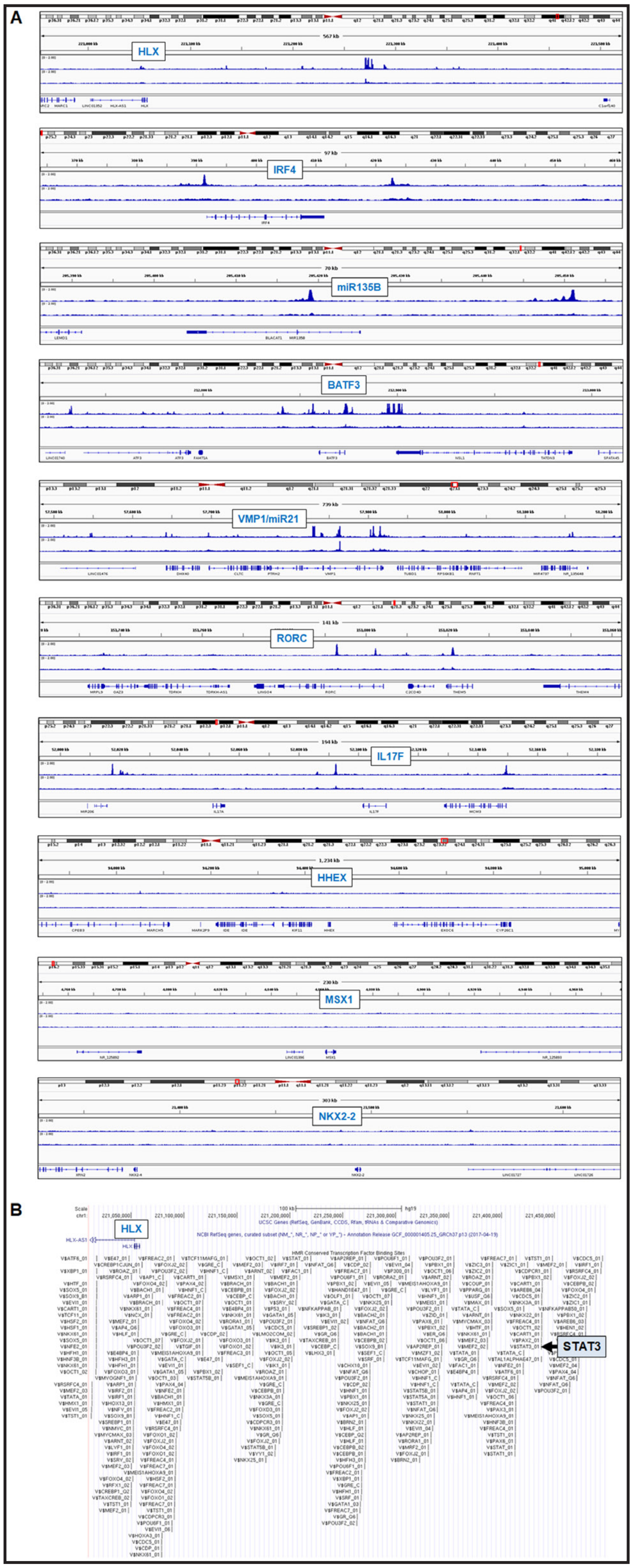

Supplementary Figure 9: ChIP-seq data for STAT3 in ALCL cell line SU-DHL-1. (**A**) ChIP-seq data were visualized using IGV as shown for the loci of HLX, IRF4, miR135B, BATF3, VMP1/miR21, RORC, IL17F, HHEX, MSX1, NKX2-2. STAT3 binding is indicated in the control (upper blue line) and after crizotinib treatment (lower blue line), showing reduced STAT3 interaction. (**B**) For the locus of HLX potential TF binding sites are indicated as obtained from the genome browser UCSC (www.genome.cse.ucsc.edu). The data correspond to release HG19.

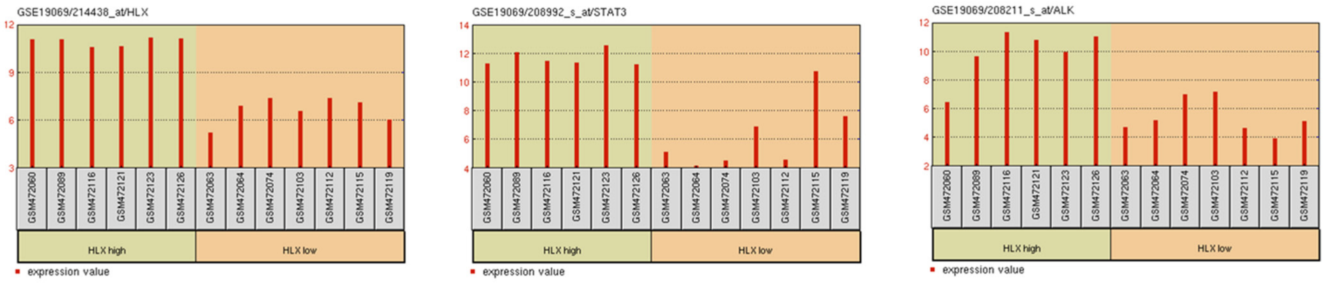

Supplementary Figure 10: Correlated gene expressions of HLX in ALCL. Selected ALCL patients expressing high and low levels of HLX were analyzed using GEO online tool GEOR. The data indicate significantly correlated expression of HLX, STAT3 and ALK in ALCL patients (dataset GSE19069).

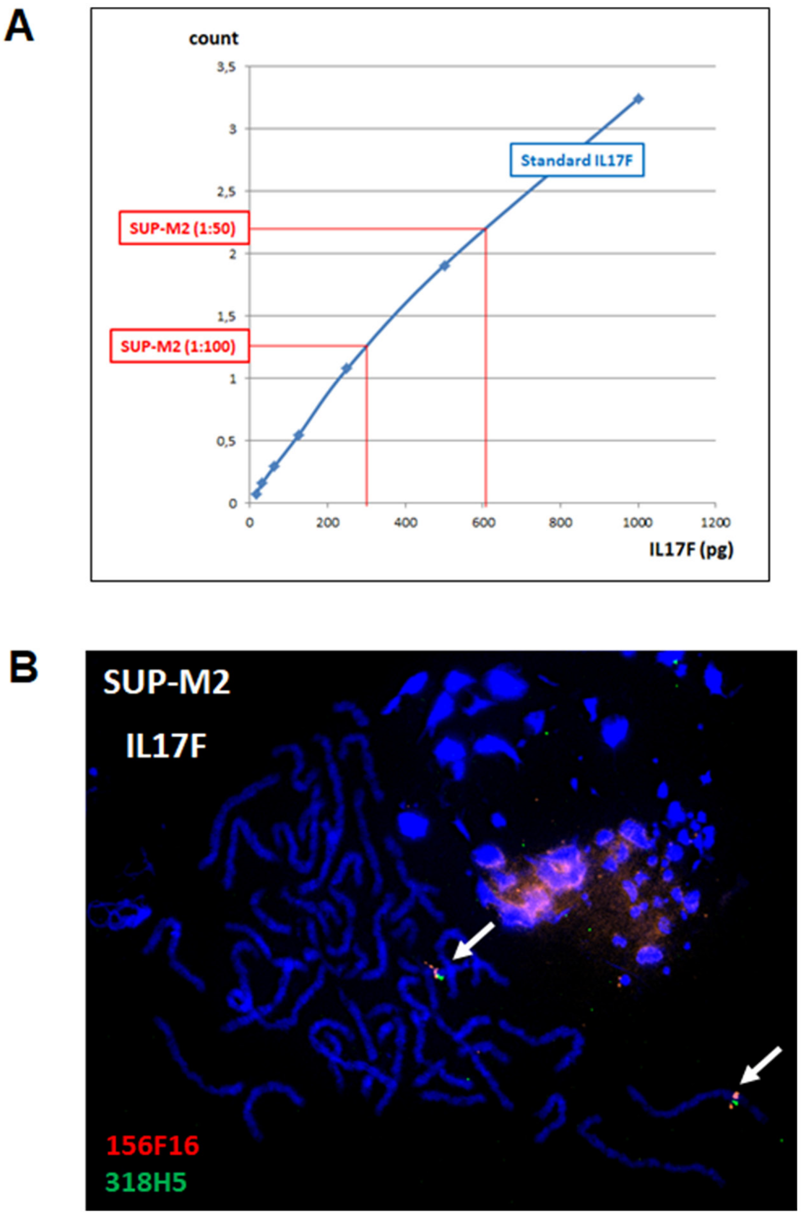

Supplementary Figure 11: FISH analysis of IL17F in SUP-M2. (**A**) ELISA analysis quantified IL17F protein in the supernatant of SUP-M2, indicating about 30 ng/ml. (**B**) FISH analysis using BAC probes RP11-156F16 (labeled in red) and RP11-318H5 (green) demonstrates absence of chromosomal translocation at the loci of IL17F (arrows). The chromosomes were counterstained using DAPI (blue).

A **MGA mutations in ALCL cell lines**

Cell line	Gene position	Alteration	Protein position	Alteration
DEL	2327	A>T	716	T>S
SR-786	2327	A>T	716	T>S
SR-786	3186	C>A	1002	A>E
SR-786	3989	T>C	1270	C>R
SU-DHL-1	2327	A>T	716	T>S
SUP-M2	2064	C>A	628	P>Q
SUP-M2	2327	A>T	716	T>S
SUP-M2	4748	C>G	1523	P>A

B

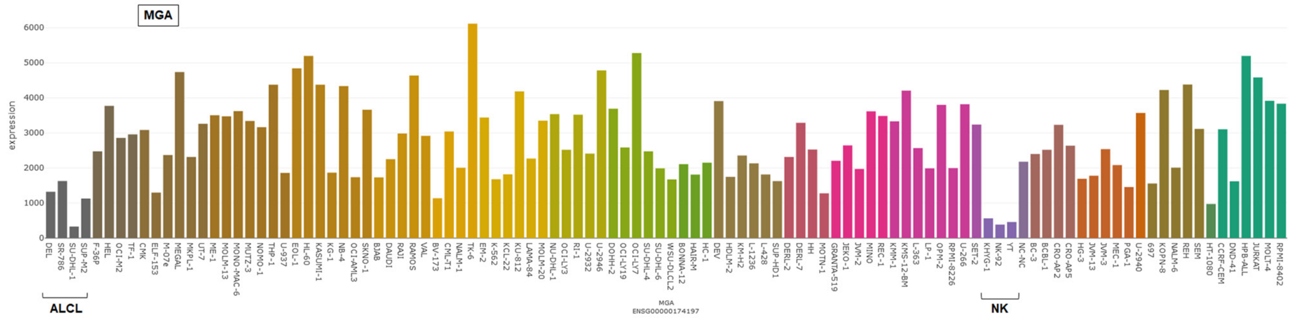

C

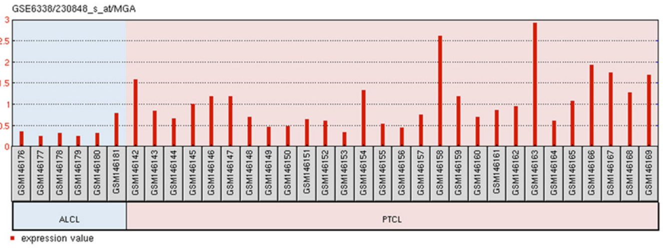

D

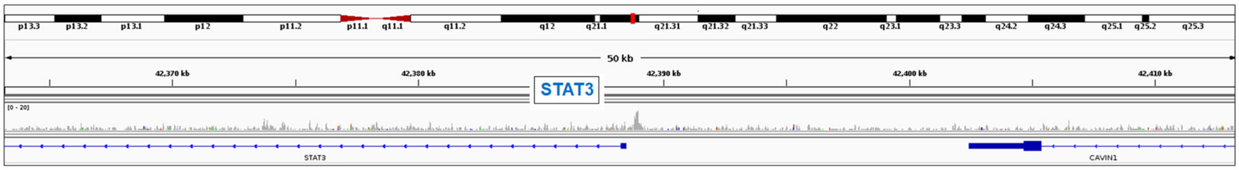

Supplementary Figure 12: Analysis of MGA. (**A**) List of MGA mutations in ALCL cell lines indicating position and alteration. (**B**) MGA expression in 100 hematopoietic tumor cell lines according to the LL-100 transcriptome dataset (E-MTAB-6006). (**C**) MGA expression in ALCL and PTCL patients (dataset GSE6338) shows significantly correlated activity as analyzed by GEO2R. (**D**) ChIP-seq data of MGA (dataset E-MTAB-6006) indicate binding at the transcriptional start site of STAT3.

A

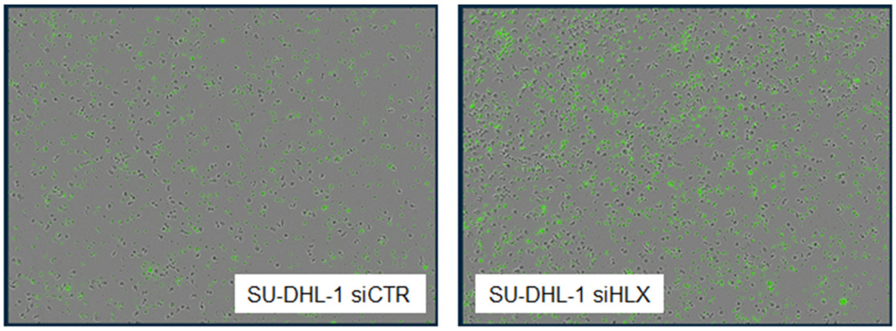

20h, apoptotic cells are indicated in green

B

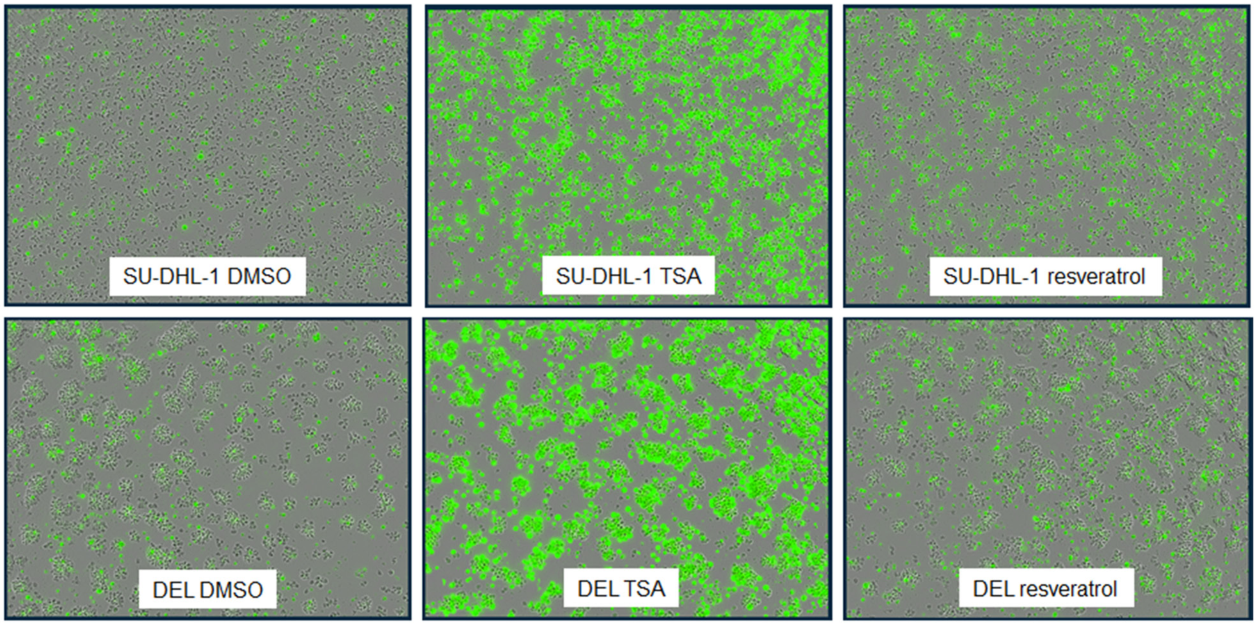

18h, apoptotic cells are indicated in green

Supplementary Figure 13: Live-cell analyses of ALCL cell lines. (**A**) Live-cell imaging pictures of SU-DHL-1 treated for siRNA-mediated knockdown of HLX after 20 hours. Apoptotic cells are indicated in green. (**B**) Live-cell imaging pictures of SU-DHL-1 (above) and DEL (below) treated with deacetylase-inhibitors TSA and resveratrol after 18 hours. Apoptotic cells are indicated in green.

Supplementary Table 1: Gene expression data of ILCs (dataset GSE112591). See Supplementary Table 1

Supplementary Table 2: Gene expression data of ILCs (dataset GSE124474). See Supplementary Table 2

Supplementary Table 3: Gene expression data of ILCs (dataset GSE90834). See Supplementary Table 3

Supplementary Table 4: Gene expression data of ILCs (dataset E-MTAB-8494). See Supplementary Table 4

Supplementary Table 5 : Expression profiling analysis of HHEX target genes in SU-DHL-1 (dataset GSE146391). See Supplementary Table 5

Supplementary Table 6 : Expression profiling analysis of HLX target genes in SU-DHL-1 (dataset GSE146391). See Supplementary Table 6
